# *In silico* structural elucidation of RNA-dependent RNA polymerase towards the identification of potential Crimean-Congo Hemorrhagic Fever Virus inhibitors

**DOI:** 10.1038/s41598-019-43129-2

**Published:** 2019-05-02

**Authors:** Muhammad Usman Mirza, Michiel Vanmeert, Matheus Froeyen, Amjad Ali, Shazia Rafique, Muhammad Idrees

**Affiliations:** 1 Department of Pharmaceutical and Pharmacological Sciences, Rega Institute for Medical Research, Medicinal Chemistry, University of Leuven, Leuven, Belgium; 2 Center for Research in Molecular Medicine (CRiMM), The University of Lahore, Lahore, Pakistan; 3 Department of Genetics, Hazara University, Mansehra, Khyber Pakhtunkhwa, Pakistan; 4 Centre for Applied Molecular Biology (CAMB), University of the Punjab, Lahore, Pakistan; 5 Vice Chancellor, Hazara University Mansehra, Mansehra, Pakistan

**Keywords:** Computational biology and bioinformatics, Virtual screening, Antivirals

## Abstract

The Crimean-Congo Hemorrhagic Fever virus (CCHFV) is a segmented negative single-stranded RNA virus (−ssRNA) which causes severe hemorrhagic fever in humans with a mortality rate of ~50%. To date, no vaccine has been approved. Treatment is limited to supportive care with few investigational drugs in practice. Previous studies have identified viral RNA dependent RNA Polymerase (RdRp) as a potential drug target due to its significant role in viral replication and transcription. Since no crystal structure is available yet, we report the structural elucidation of CCHFV-RdRp by in-depth homology modeling. Even with low sequence identity, the generated model suggests a similar overall structure as previously reported RdRps. More specifically, the model suggests the presence of structural/functional conserved RdRp motifs for polymerase function, the configuration of uniform spatial arrangement of core RdRp sub-domains, and predicted positively charged entry/exit tunnels, as seen in sNSV polymerases. Extensive pharmacophore modeling based on per-residue energy contribution with investigational drugs allowed the concise mapping of pharmacophoric features and identified potential hits. The combination of pharmacophoric features with interaction energy analysis revealed functionally important residues in the conserved motifs together with *in silico* predicted common inhibitory binding modes with highly potent reference compounds.

## Introduction

The Crimean-Congo hemorrhagic fever (CCHF) is a devastating viral infection with an extremely high case-to-fatality ratio (ranging from 5–30%, and 50–80% during epidemic events)^1^ caused by a tick-borne Crimean-Congo hemorrhagic fever virus (CCHFV) from the genus *Nairovirus* (Family *Orthonairovirus*, order *Bunyaviridae*)^2–4^. Being a classically tick-borne disease, the genotypic classification of CCHFV yielded supportive epidemiological data classifying the *Hyalomma* tick as an important carrier^5^. Messina *et al*.^6^ analyzed human CCHF occurrence data up to 2015 (containing 1,721 geo-positioned occurrences) and reported a full description of its zoonotic niche and the immediate risk to humans. With 10 to 50% mortality and widespread distribution, this arbovirus becomes a substantial concern in case of human-to-human transmission^7,8^. CCHFV infection starts with non-specific symptoms (from rapid onset high-grade fever, fatigue, myalgia following vomiting and diarrhea) and progresses to a severe disease state with gastrointestinal and cerebral hemorrhages^2,9,10^. As the disease progresses, CCHFV is observed in the spleen, pulmonary, cardiac, and intestinal tissues in fatal cases in humans^11^.

The CCHFV viral genome is composed of three single-stranded negative-sense RNA segments referred to as small (S) (~1.6 kb), medium (M) (~5.4 kb) and large (L) (~12.1 kb) segments which encode viral nucleoprotein (NP), the glycoprotein precursor (GPC) and L protein respectively, all of which have complementary 3′ and 5′ termini^12^. The L protein fragment contains the viral RNA-dependent RNA polymerase (RdRp) domain. Together with viral RNA (vRNA), NP and RdRp form the genomic ribonucleoprotein complexes (RNPs)^13^, CCHFV viral entry, transcription/replication cycle, glycoprotein maturation and viral assembly have been recently and extensively reviewed^10,14,15^. In short, CCHFV enters host cells by endocytosis after engaging cell surface receptors using mature glycoproteins (Gn and Gc) and releases the genome. After membrane fusion, the genomic segments are uncoated and transcribed by the L protein into viral mRNA and subsequently translated into NP and L proteins. The endoplasmic reticulum-associated ribosomes translate the GPC. Fragments of newly synthesized NP and L protein are used for replication of genomic RNA, forming RNP. Inside ER and Golgi bodies, the newly CCHFV particles undergo processing and maturation, followed by virion release in Golgi-derived vesicles via exocytosis^10,16^.

*Nairovirus* RdRps species share a characteristic right-handed structure with three subdomains (finger, palm, and thumb) indicative and essential for catalytic activity^17,18^. In all *Nairovirus*, the RdRp domain maintains six characteristic conserved motifs including PreA/F, A, B, C, D, and E in the central region^19–22^. Most of these motifs are located in the palm subdomain and define the formation of the active site^21^.

Despite a high mortality rate, no licensed antiviral drugs or vaccines are currently available for CCHF^23^. Efforts have been made in vaccine development^24,25^ with Bulgarian vaccine^26^, DNA vaccine^27^ and the recent NP-based vaccine^28^. Moreover, a large multi-national consortium, CCHFVaccine, is supporting the development of a CCHFV vaccine which would be a major tool to limit an outbreak (http://www.cchfvaccine.eu/). Broad-spectrum ribavirin has demonstrated antiviral activity when administered in early infection^29,30^ and also indicated *in vitro* efficacy in infant mouse models^31,32^. Favipiravir (T-705) has reported increased activity compared to ribavirin^33^ with additional activity against two distinct strains of CCHF virus in mouse models^34^. A screen of candidate nucleoside analog compounds identified 2′-deoxy-2′-fluorocytidine as more potent than ribavirin (200-fold) and T-705 (17-fold)^35^ and has very recently been reported to be a broad-spectrum inhibitor of *Bunyaviruses* after *in vitro*/*in vivo* studies^36^. Other experimental therapeutics with no evidence in humans include two repurposed FDA molecules, chloroquine (IC_50_ 39.4 to 28.1 μM) and chlorpromazine (IC_50_ 10.6 to 15.8 μM). MxA (interferon-induced GTPase)^37^ and ISG20 (interferon-induced exonuclease)^23,38^ showed direct activity against CCHFV^39^.

For antiviral drug discovery purposes, druggable viral targets and host proteins are of main interest^40–44^. CCHFV has a complex genome with multiple proteins involved in processes ranging from virus entry into host cells to viral replication and suppression of the host immune system^10^. RdRp has a pivotal role in the replication process and is involved in the crucial association of viral RNA to make RNP complexes^15^.

CCHFV-RdRp core domain of L protein was selected as a target for which no licensed drug has been reported to date. Besides evolutionary conserved motifs in the core region, RdRps have channels/tunnels that connect their catalytic center with the protein surface and emerge as potential targets for developing anti-viral inhibitors^45–47^. The same paradigm is found in the inhibitor design against many lethal viruses such as *Japanese encephalitis virus* (JEV)^48^, *Zika virus* (ZIKV)^49–52^, *Dengue virus* (DENV)^53–55^, *West nile virus* (WNV)^56^, HCV^57–59^ and most of drugs targeting *Ebola* polymerase L (EBOV)^60^.

Despite the profound antiviral activity of broad-spectrum antivirals activity against CCHFV^31–35^, the mode of action of T-705 and ribavirin remains suggestive. In accordance with other negative strand viruses, studies showed inhibition of viral RNA polymerases by incorporation of T-705-ribofuranosyl-5′-triphosphate^61^ and ribavirin-triphosphate^62^ into nascent RNA strands causing lethal mutagenesis^63,64^.

Computational methods provide both an alternative and a supplement to tiresome high-throughput screening^65–68^, which gave researchers the opportunity to hasten, facilitate and innovate the effectiveness of the overall drug discovery process^69–73^. Integrated virtual screening methods, including structure-based (SBVS) and ligand-based virtual screening (LBVS), have identified active antiviral compounds against viral epidemics such as *influenza* virus^74^, EBOV^41,75,76^ DENV^77–80^ and ZIKV^52,81^, while others reported the aid of molecular dynamics (MD) simulations and binding free energy calculations in search for potent antivirals^50,78,82–88^ and investigating drug resistance mechanisms^82,86,89–91^.

The crystal structure of CCHFV-RdRp has not yet been made available and can be made possible through *ab initio* methods^92,93^. Recent advances in homology modeling have proven their effectiveness as an alternative^94,95^ with retrospective analysis validating the usefulness of homology modeling in SBVS^96–99^. Here, we report an optimized cost and time efficient strategy starting from an extensively refined homology model of CCHFV RdRp as a potential druggable target, followed by step-wise pharmacophore-based virtual screening and all-atom backbone molecular dynamics simulation of potential hits.

## Materials and Methods

### Homology modeling and refinement using Molecular Dynamics (MD) simulations

Homology modeling plays a significant role in the drug discovery process^94^ with current efforts resulting in models with unprecedented accuracy^95,100^ even with low sequence identity to the template^101–105^. Because of the absence of a crystal structural of CCHFV-RdRp, homology modeling was necessary for target structure elucidation. To implement this, CCHFV-RdRp protein sequence was retrieved from the RefSeq database (NCBI Reference Sequence YP_325663). A PSI-BLAST^106^ search resulted in templates with less than 20% sequence identity with the target of interest. Therefore, an extensive comparative homology modeling protocol was applied in four steps as follows: (1) prior to homology modeling, careful consideration was taken into account for biologically suggestive template selection. For this, various software with different built-in algorithms were used to identify all possible templates including HHpred^107^, LOMETS (LOcal MEta-Threading-Server)^108^,MUSTER (MUlti-Source ThreadER)^109^, ITASSER (Iterative Threading ASSEmbly Refinement)^110^, RaptorX^111^ and SWISS MODEL^112^. Combination of different features of these tools provides an unbiased set-up for template identification.

(2) The models generated from identified templates were structurally compared with respective templates using TM-align^113^, FATCAT^114^ and MATRAS^115^ to identify similarities and differences which result in template selection used to model structural elements. TM-align evaluates optimal structural similarity by TM-score (score > 0.5 indicates the two structures likely to have the same fold in SCOP/CATH). MATRAS compares secondary structural elements and classifies structures based on SCOP superfamily and FOLD. FATCAT allows flexible protein structure comparison to evaluate structural similarity by P-value (structure pairs with P-value < 0.05 indicate significantly similar). Based on several structural attributes between template and model, including comparable secondary structural elements, presence of similar folds, RMSD of equivalence residues, C-alpha backbone RMSD, best models were selected together with the most fitted template.

(3) The final model was built using a restrained-based approach in MODELLER.v9.1^101^ with the most-fitted template, together with the secondary structural information obtained by manual curation after superimposition between all generated models (step 2) and template. The extracted spatial secondary structure restraints were implemented to model the final structure using the secondary structure module of MODELLER. Target-template alignment was adjusted to reduce the number of misaligned residues.

(4) The models selected in step 2 and 3 were refined by 20 ns MD simulation run and evaluated (before/after MD) via Molprobity (MP) metrics ranked by CASP. Based on the MP-score, the final model was selected, followed by extensive 100 ns MD simulation to check the stability of all backbone atoms. A large ensemble of 1000 snapshots generated by an MD trajectory was used to select the representative conformation from a largest cluster based on RMSD cut-off of ~1 Å. This representative conformation CCHFV-RdRp was further selected to perform modeling studies.

The AMBER16 package using the AMBER ff99SB force field^116^ was used for unrestrained molecular dynamics simulations. After a stepwise energy minimization and equilibration protocol (as described in a previous studies^40,117^), the solvated system with explicit TIP3 water molecules was submitted to a production run of 100 ns at constant temperature (300 K) and pressure (1 bar) (detailed protocol in supplementary information).

The MD simulation complete trajectory was collected with a time interval of 2 ps and analyzed using the CPPTRAJ module^118^. Backbone dynamics were analyzed for both reference template and generated model over a period of 100 ns The most representative conformation of model was obtained as explained in step 4. Later the modeled structure was evaluated through Ramachandran plots generated by MolProbity.

### Binding site analysis

A variety of diseases have been associated with the mechanism of involved proteins and their interaction with small molecules^119^. Identification and characterization of active sites of target proteins using *in silico* methods are of prime focus to researchers these days^41,120^. For the identification of active sites, analytical tools are currently being applied since the number of known protein structures is growing fast^121–125^. To further support the identification of the active site and its residues, COACH meta-server^126^ and 3DLigSite^127^ were used. In these methods, the protein function database with ligand-binding templates, BioLiP, is used^128^. Additionally, the complete protocol for COACH combines the results of several other programs including COFACTOR, Con-Cavity, and FINDSITE resulting in the generation of highly accurate protein-ligand binding site predictions. 3DLigSite incorporates ligand-bounded complexes similar to the query and superimposes these onto the model to predict the binding site.

### Molecular docking of potent inhibitors

Small molecules including ribavirin, arbidol and T-705 were selected to be docked at the predicted binding site of CCHFV-RdRp. All these inhibitors were documented to exhibit CCHFV inhibition^31,129,130^ *in vitro* and *in vivo* within a more broad range of RNA viruses^131–134^. The optimized and refined CCHFV-RdRp model was minimized and prepared according to the standard protocol of AutoDock (AD) Vina as described elsewhere^41,43^. Docking grid was set up around the predicted binding site residues covering the conserved structural motifs to reduce the search space for ligand optimization^135^. The antechamber module from AMBER16^116^ was used to generate atomic partial charges for the test compounds. The docked complexes were further subjected to MD simulations over a period of 10 ns using the protocol above. As a proof of concept, ribavirin 5′-triphosphate (RTP) and the recently identified 2′-Fluoro-2′-deoxycytidine (2′-FdC) were docked as reference compounds^35,36^. RTP is the active form of ribavirin^136^ and 2′-FdC was reported to be 200-fold more potent compared to ribavirin and 17-fold more potent than T-705 against CCHFV,

### MM/GBSA binding free energy calculations and Per-residue decomposition analysis

The Molecular Mechanics/Generalized Born Solvent Area (MM/GBSA) method implemented in AMBER 16.0 was employed to estimate the binding affinity of the test compounds. To gain rational insights into the different binding modes, an energy decomposition analysis was performed examining the energetic contribution of each residue interacting with the different compounds. The MM/GBSA approach is well documented in binding free energy calculations^137^ for antiviral inhibitors^138,139^.

### Pharmacophore model generation and Virtual screening workflow

The reported inhibitors ribavirin, arbidol and T-705 were simulated over a period of 10 ns at the predicted binding site of CCHFV-RdRp to get the most stable conformations of bound ligands. Subsequently, virtual screening process was carried out in several steps starting with the generation of a structure-based pharmacophore model and library generation. For this, the pharmacophore residues were selected based on the energy decomposition profile with reported anti-viral drugs. The constructed model was then added to Ligandscout, to screen the mcule database^140^ and subjected to the Lipinski’s Rule of Five criterium^141^. Following this, the generated compound library was further reduced through pharmacokinetics (PK) and pharmacodynamics (PD) filters. Subsequently, drug safety was evaluated through series of PAINS (Pan Assay Interference Compounds) filters^142^ and Brenk structural alert^143^ by detecting unwanted toxicophores iv) The screened compounds were then finally docked into the explicit binding site of the refined and optimized CCHFV-RdRp using AD Vina. After screening, the ligand with highest binding affinity was selected for further processing. v) The apo conformation of CCHFV-RdRp was then simulated for a period of 10 ns and four snapshots were taken every 2.5 ns (2.5 ns, 5 ns, 7.5 ns and 10 ns), thereby taking the target flexibility into account^144^. To finally dilute the compound library, the screened compounds from step (iv) were docked in these four time-dependent conformations. Hits were selected based on binding energy convergence between all four time-dependent conformations of CCHFV-RdRp, and further subjected to 10 ns MD simulations followed by per-residue energy decomposition analysis, as described in molecular dynamics simulation protocol section.

## Results

### Structure prediction of CCHFV-RdRp

A wide range of viruses encodes the RdRps and have a vital role in replication and transcription of vRNA^15^. All crystallographic resolved RdRps structures display a similar right hand-like structure with three representative subdomains: finger, palm, and thumb subdomains^17^. Before structure prediction, high-resolution RdRp structures were examined from segmented negative single-stranded RNA viruses (−ssRNA, order *Bunyaviridae*) including *La Crosse orthobunyavirus* (LaCV) (PDB ID: 5amr, 5amq) and Influenza A and B (4wsb, 4wrt) (extensively reviewed elsewhere^15,145^). These findings revealed the similar overall architecture of L-protein and conserve RdRp core including the conserved finger, palm and thumb subdomains with conserved six structural motifs which are essential for polymerase function^47^ **(**Supplementary Fig. S1**)**. Despite the low sequence similarity (~19% identity, ~33% similarity) outside the endonuclease domain^146^ and conserved structural motifs^20^, the recently resolved LaCV L- protein fragment comprising 1750 residues (77% of 2263 residues referred to as L_1750_) revealed a conserved overall architecture with a hetero-trimeric complex of Influenza B virus (formed by the PA, PB1, and PB2 subunits). The RdRp active site is connected with four positively charged tunnels which maintain nucleotide triphosphates entry, nascent strand exit, template entry and template exit, surrounded by conserved residues in *orthobunyavirus* polymerases^21,147^. The multiple sequence alignment of the central region of RdRp of *Bunyaviridae* genera **(**Fig. S1**)** highlighted all motifs present in the fingers and palm subdomain to be well conserved including premotif A/F (**K**x**Q**x_5_**R**…**K/R**x_6_**E**), motif A (**D**x_2_**KW**), B (**QG**x_5_**SS**), H (**K**ELIL in LaCV), signature motif C (**SDD**), D (KKT in LaCV), E (**E**x_2_**S**x**F**). This pinpoints to the potential of exploiting this evolutionary conserved RdRp core architecture with other related segmented (−) ssRNA viruses to predict their functional and structural features even with a low sequence homology.

Following this, possible templates were identified against CCHFV-RdRp core domain of L-protein using the HHpred server. As expected, HHpred indicated less than 20% identity with the top hits, which included RdRps of LaCV (5AMR) and Influenza B (4WSB, 4WRT) and C virus (5D98).

HHpred uses HHblits, profile-profile and HMM-HMM method, which are able to detect the homologous relationship in evolutionarily related proteins with less than 20% identity, based on conserved structural elements^148^. All template hits against CCHFV-RdRp revealed homologous to each other (i.e., members of same SCOPe superfamily) and classified in the same class of (−) ssRNA (segmented) viruses including *Bunyaviridae* and *Orthomyxoviridae*.

A different set of input parameters, from local to global alignment and HHblits to PSI-BLAST revealed the same results and therefore suggested CCHFV-RdRp to be globally homologous to the putative template hits. Together with an estimated probability of hits (exactly 100% query cover), significant E-value (ranged from 9.8 e^−47^ to 2.8 e^−35^ between all RdRps hits) and presence of conserved structural motifs having similar folds suggested these hits as potential templates for homology modeling of CCHFV-RdRp. To rationalize the modeling approach in an unbiased manner, comparative homology modeling protocols were employed using I-TASSER, SWISS-MODEL, LOMETS, RAPTORX and MUSTER which estimate the biological connection of query sequence without the information of any template. Strikingly, all these servers predicted RdRp domains of L_1750_ (5AMQ) and Influenza A, B and C (4WRT, 4WSB, 5D98) viruses as best templates and recognized identities between 14–20% with a query coverage up to 100% (Supplementary data - Tables S1, S2). The final template-guided model was build using MODELLER (PDB ID: 5 amq, 4wrt, and 4wsb as templates) by taking into account the structural features obtained from the superimposition of modeled structures with the most fitted template. Structural alignment with respective templates using TM-align, FATCAT and MATRAS servers indicated 5amq.A as most favorable template (Supplementary data). The best models from all servers were refined through 20 ns MD simulations and evaluated through Molprobity MP-score (before and after 20 ns production run) tabulated in Table 1. Based on the MP-score, the best CCHFV-RdRp model was generated through MODELLER (MP-score after refinement: 1.44 with 99^th^ percentile) which was further refined through 100 ns MD simulations to optimize overall geometry and to remove clashes in geometry for later analysis.

**Table 1:** Molprobity evaluation before and after 20 ns MD refinement of CCHFV-RdRp models created from different programs.

Programs implemented	Template	MP-score**		Clash-score*		Rot-out (%)		Rot-fav (%)		Ram-out (%)		Ram-fav (%)	
		before	after	before	after	before	after	before	after	before	after	before	after
I-TASSER	5amq.A	3.91	1.87 (82^nd^)	160.92	1.49 (99^th^)	5.64	3.31	85.33	89.92	4.26	2.06	86.23	88.68
LOMETS	5amq.A	3.7	1.79 (86^th^)	138.76	1.29 (99^th^)	5.33	2.45	84.99	87.76	4.16	2.95	88.13	86.01
RAPTORX	5amq.A	3.73	1.88 (81^st^)	138.6	1.55 (99^th^)	5.43	3.38	85.12	90.09	4.21	2.3	89.26	88.63
MUSTER	5amq.A	3.85	1.7 (89^th^)	150.44	1.16 (99^th^)	5.36	2.33	82.7	89.86	5.05	2.3	85.25	88.31
MODELLER^+^	5amq.A	3.4	1.44 (99^th^)	80.7	0.65 (99^th^)	4.29	1.82	86.04	90.4	3.42	1.68	90.16	91.89

^*^Clash score is the number of serious steric overlaps (>0.4 Å) per 1000 atoms.

**MolProbity score combines the clashscore, rotamer, and Ramachandran evaluations into a single score, normalized to be on the same scale as X-ray resolution. (100^th^ percentile is the best among structures of comparable resolution; 0^th^ percentile is the worst.

^+^Final model by MODELLER was made using 5 amq.A as most fitted-template based on conserved structural attributes obtained from multiple 3D structural alignments of all models (built from other programs) with 5 amq.A.

### Overall homology model of CCHFV-RdRp

**Figure 1:**
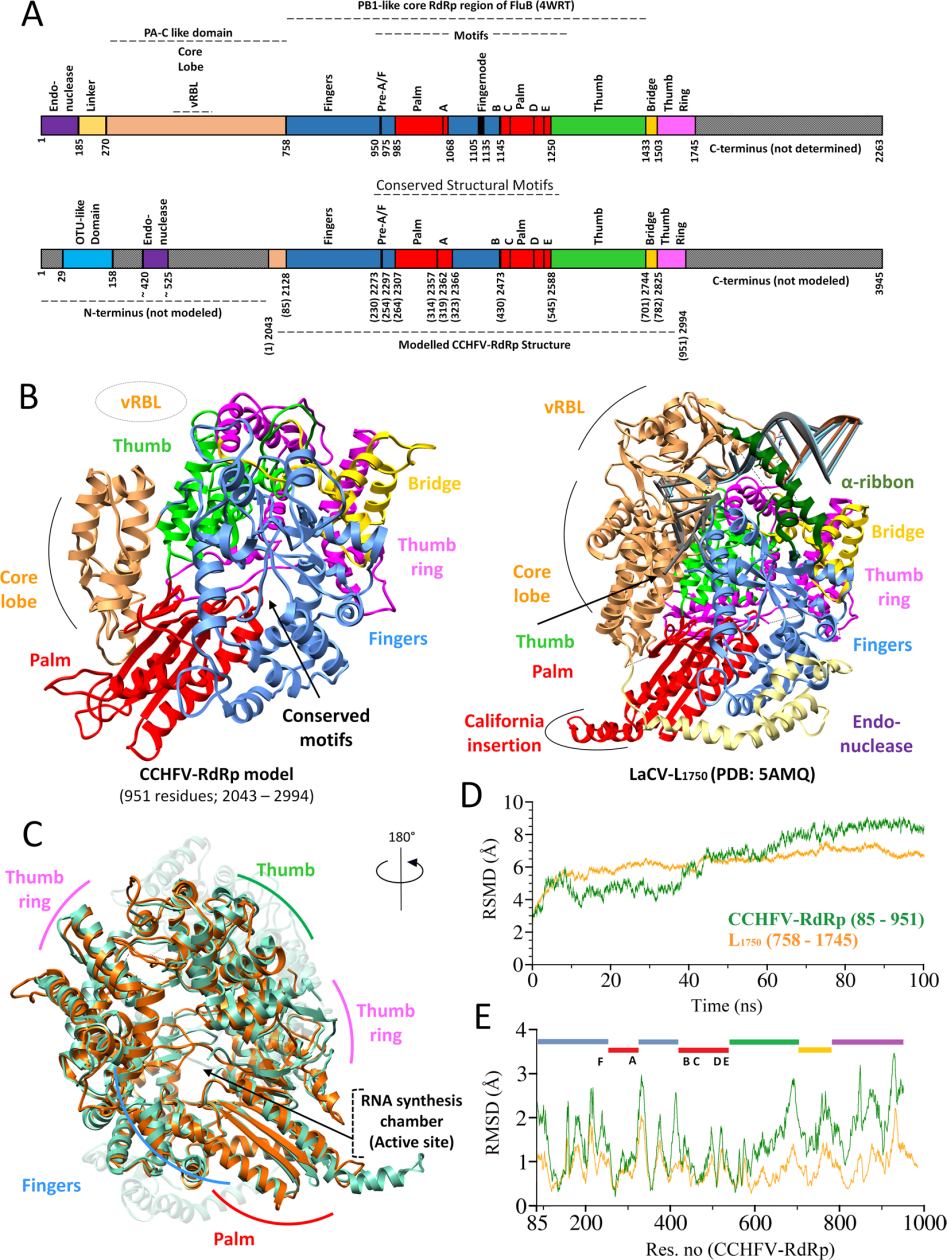
Overall homology model of CCHFV-RdRp. (**A**) Linear schematic representation of domain architecture of CCHFV-RdRp (modelled 2043–2994; 951 residues) (top) aligned to L_1750_ (bottom), with structurally and functionally conserved domains similarly colored (**B**) Structural representation of same pose of CCHFV-RdRp model (left) with L_1750_ (right), elaborating the similarly colored structural homologous features including canonical fingers, palm and thumb subdomains along with other domains (**C**) Structural superimposition of CCHFV-RdRp (orange) and L_1750_ (green) highlighting the similar configuration of thumb and fingers subdomains by their positions in space relative to the palm subdomain. The conformation is rotated to display the conserved RNA synthesis chamber (active site). (**D**) Root Mean Square Deviation (RMSD) of CCHFV-RdRp model (green) in Angstrom (Å) during 100 ns simulations together with the reference L_1750_ structure. (**E**) Root Mean Square Fluctuation (RMSF) of all residues of CCHFV-RdRp model along with the reference L_1750_ structure during MD simulation and represented domains are highlighted with their respective colors (as in (**A**)).

The overall homology model of CCHFV-RdRp (residues 2043–2994; renumbered 1–951 in homology model) was superimposed on L_1750_ protein (5AMQ)^21^ and compared the RdRp core subdomains. The central PB1-like RdRp region of L_1750_ (residues 758–1433), was previously determined in Influenza polymerase (5AMQ)^147^. The modelled CCHFV-RdRp core region includes the fingers (overlapping residues 85–430), palm (overlapping residues 265–545) and thumb (residues 546–782) subdomains with conserved structural motifs exposed in the internal core RNA synthesis cavity and display the strikingly similar overall conformation of three core subdomains **(**Fig. 1A,B**)** despite the lack of significant sequence homology. Sequence homology with L_1750_ shows the endonuclease domain of CCHFV-RdRp to be located approximately ~1500 amino acids upstream from the finger domain However, model superimposition represented high structural similarity with the larger α-helical core lobe of L_1750_ (starting from ~ 85 aa upstream from the finger domain of CCHFV-RdRp) **(**Fig. 1A**)**. The superimposition of CCHFV-RdRp homology model and L_1750_ revealed overall structural similarity, with RMSD values of modelled secondary structural elements (Cα-backbone atoms) of 1.32 Å, 1.41 Å and 1.79 Å for finger, palm and thumb subdomains respectively. Other conserved features include the C-terminal Bridge (a helical bundle representation from residues 701–782) which closes the circular architecture of the polymerase core around the internal RNA synthesis chamber, analogous to L_1750_^21^ **(**Fig. 1A,B**)**. This characterized the similar conformations of important secondary structural elements such as a partially ordered “α-ribbon” (residues 847–905 in L_1750_) in the finger domain, California insertion (residues 1021–1044 in L_1750_) in palm domain **(**Fig. 1B**)**, a unique exposed helical structure in *orthobunyavirus*^21^ and priming loop (residues 1402–1422 in L_1750_) at the C-terminal end of thumb domain. Reverse template comparison versus structures in the protein databank (PDB) was performed by submitting modelled structure on profunc server^149^ to further support the structural similarity. This server uses Jess, a fast and accurate 3D-structure search algorithm, to scan auto-generated templates from the query structure against representative structures in PDB. As expected, the reverse template search resulted in RdRp domains of L_1750_ (5AMQ) and Influenza B (4WRT) among the best hits in terms of E-value (4.48 e^−09^, 1.77 e^−08^) and structural similarity (87.9%, 81.8%) (Table S3). The RMSD trajectory plots represent the overall-backbone structure stability of core RdRp subdomains of CCHFV (residues 85–951, in green) compared to L_1750_ structure (residues 758–1745, in orange) including the central PB1-like RdRp region of L_1750_ (residues 758–1433) and C-terminal Bridge and thumb ring domains **(**Fig. 1D**)**. CCHFV-RdRp displayed a similar RMSD value (~3 Å) with the template. The RMSD trajectory of L_1750_ (residues 758–1745) remained converged from 15 to 100 ns, while backbone Cα-RMSD of CCHFV-RdRp fluctuated in the start and later converged. The slightly higher RMSD in the modelled structure, was presumably caused by protein expansion during simulation to attain a more stable conformation. To investigate the mobility of individual residues, root-mean-square-fluctuations (RMSF) were calculated as shown in Fig. 1E. RMSF plot of Cα-backbone atoms of CCHFV-RdRp model was analyzed together with L_1750_ to observe the flexibility in the overall structure. Notably, RMSFs of individual RdRp core domains, including fingers, palm, and thumb revealed a similar trend compared to L_1750_, especially in regions with conserved motifs. Small C-terminal peaks indicated a more open structure due to partially modeled thumb-ring domain of CCHFV-RdRp as compared to L_1750_ (Fig. 1D). The final CCHFV-RdRp model was selected as a representative conformation obtained from the largest cluster, based on RMSD cut-off of ~1 Å, and evaluated with Molprobity. This indicated 92.12% (876/951 residues) of all residues in Ramachandran favored regions and 97.79% (929/951 residues) of all residues in allowed regions with only 1.78% (17 residues) outliers **(**Fig. S2**)**.

### Potential active site prediction of CCHFV-RdRp

**Figure 2:**
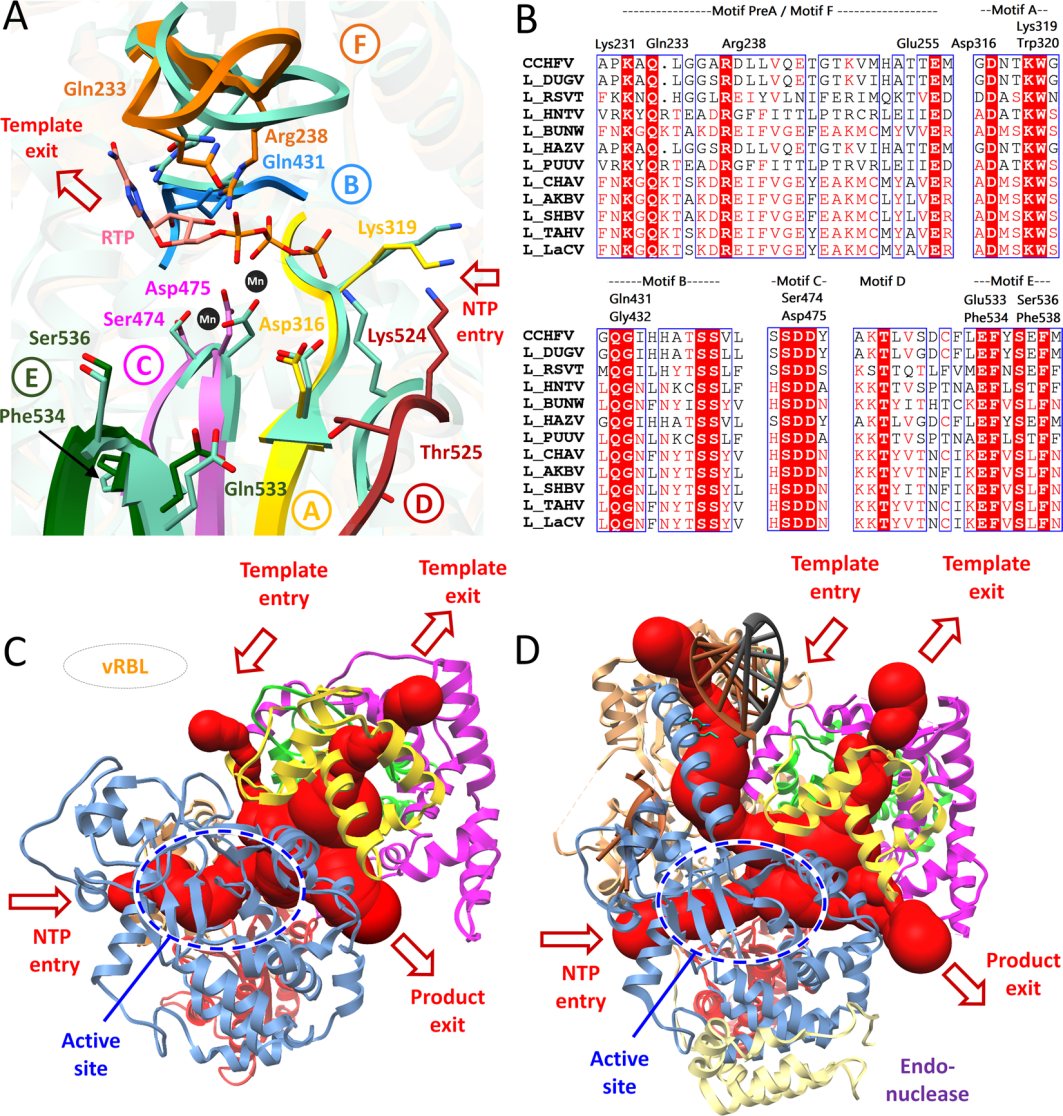
The predicted CCHFV-RdRp active site and positively charged tunnels. (**A**) The arrangement of structurally conserved RdRp motifs in CCHFV-RdRp model colored yellow, dogged blue, orchid, brown, green and orange for motifs A-F respectively, and superimposed on L_1750_ (sea green). (**B**) The corresponding predicted binding site residues are aligned through multiple structure alignment in corresponding motifs. Superposition of the polio virus elongation complex structure (PDB: 3OL8) and foot-and-mouth disease virus in complex with RTP (PDB: 2E9R) shows the positions of the catalytic divalent cations (black spheres) and RTP (salmon) (**C**) The CCHFV-RdRp model is shown (in ribbon) with four predicted positively charged tunnels (red) marked with arrows as entrance (template and NTP entry channel) and exit (template and nascent strand exit channel) tunnels calculated with MOLE2.0. The domains are same as colored in Fig. 1A. (**D**) The same representation as (**C**) for L_1750_ (PDB: 5 amq) with the 5′ and 3′ vRNA is highlighted. Domain colors are same as in Fig. 1A.

The active site cavity of CCHFV-RdRp and conserved polymerase motifs A-F accurately mimicked the L_1750_ core subdomains **(**Fig. 1C**)**. To identify potential binding site residues, COACH and 3DLigandSite were used, together with multiple sequence alignment with closely-related RdRp protein sequences^150,151^. Both servers predicted Leu234, Arg238, Asp316, Lys319, Trp320, Gln431, Gly432, Ser474, Asp475, Glu533, Phe534, Ser536 and Phe538 as common binding site residues which were shown to be conserved by the multiple sequence alignment **(**Fig. S1**)**. Interestingly, the structural superimposition with L_1750_ demonstrated all predicted binding site residues to be exclusively inside the functionally important conserved polymerase motifs of CCHFV-RdRp **(**Fig. 2A**)**. These include premotif A/F (230-KAQ**L**GGA**R**), motif A (316-**D**NT**KW**) with divalent cation binding Asp316 (Asp1060 in L_1750_), motif B (431-**QG**IHHATSS), catalytic signature motif C (474-**SD**D), and motif E (533-**EF**Y**S**E**F**) (binding site residues in bold) **(**Fig. 2B**)**. More importantly, the predicted binding site residues were found to be stable within ~1 Å fluctuation, as compared to L_1750_ **(**Fig. 1B**)**.

The modeling reliability of CCHFV-RdRp allows the prediction of entrance (template and NTP entry channel) and exit (template and nascent strand exit channel) tunnels, calculated with MOLE 2.0^152^, similar to that reported for L_1750_^21^ and influenza polymerase^147^. Figure 2C highlights the prediction of four positively charged tunnels that join together in the active site RNA synthesis chamber where conserved polymerase motifs arbitrate the template-directed RNA synthesis. The template channel entrance in the modelled structure could not be properly defined due to the unavailability of modelled viral RNA binding lobe and α-ribbon albeit minor template entrance channel similarity to L_1750_ (Fig. 2D). The NTP entrance tunnel was geometrically predicted accurately and aligned with some conserved residues as described for L_1750_ including Lys230, Arg237 (Lys956, Arg958 in L_1750_) (motif pre-A/F), Lys319 (Lys1063 in L_1750_) (motif A), and Lys525 (Lys1228 in L_1750_) (motif D) (Fig. 2C,D). The product exit tunnel is enclosed by the thumb ring (predicted from 782–951), finger and palm at opposite sides of NTP entry channel as described for L_1750_. The predicted template exit tunnel is surrounded by thumb, thumb ring and bridge (predicted from 701–782) domains and lined by conserved residues as reported for L_1750_ including Lys771, Arg772 (Lys1492, Arg1493 in L_1750_) of bridge domain, and Lys941 and Arg946 (Lys1686, Arg1690 in L_1750_) of thumb ring (Fig. 2C,D). With these assumptions, the overall structural arrangement of CCHFV-RdRp tunnels and active site prediction are in accordance with the described RNA synthesis in L_1750_^21^.

### Per-residue energy distribution-based pharmacophore model

**Figure 3:**
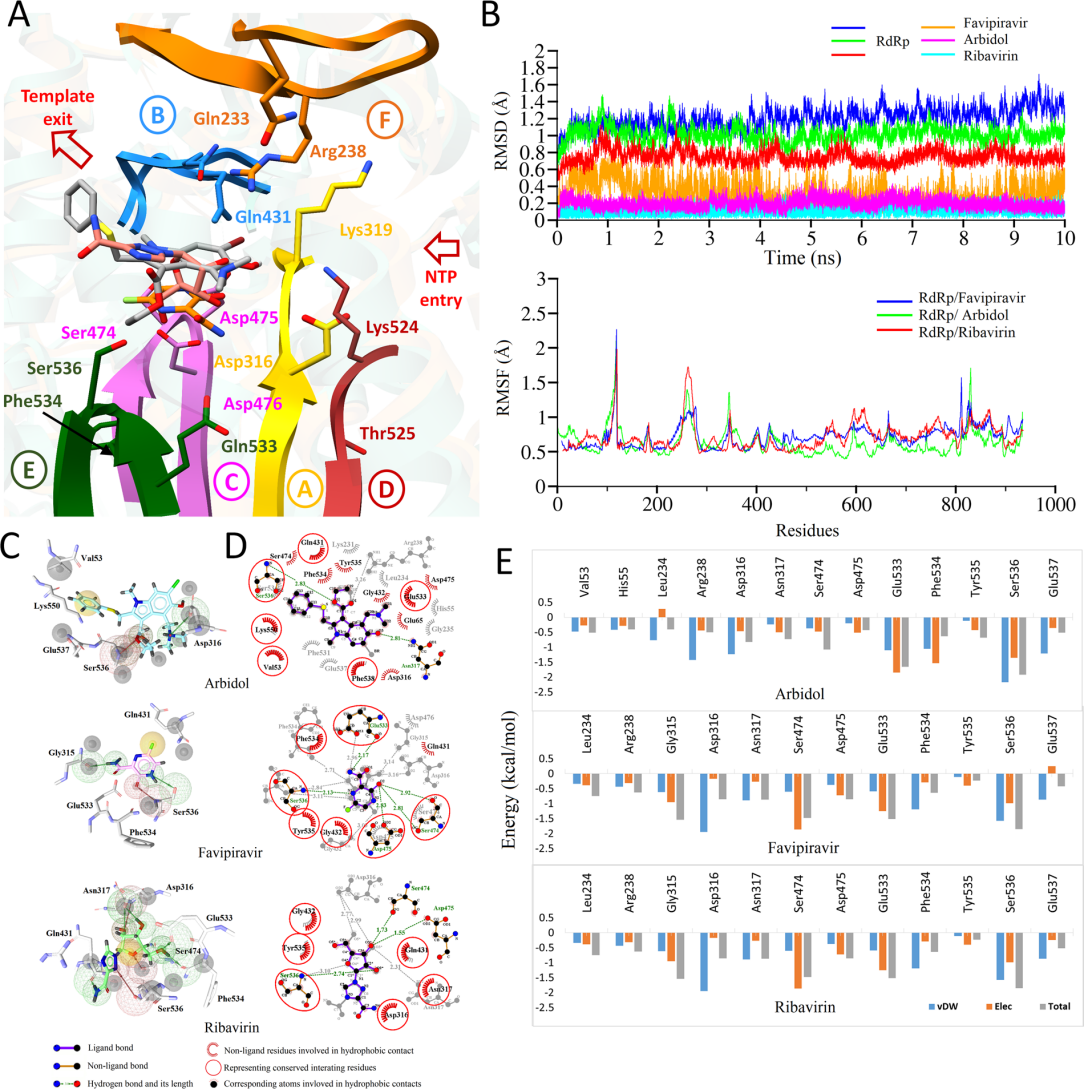
Per-residue energy distribution-based pharmacophore model. (**A**) MD simulated conformations of ribavirin (salmon), arbidol (grey) and favipiravir (orange) in sticks, inside the predicted binding pocket of CCHFV-RdRp. The arrangement of motifs and colors are same as in Fig. 2A. (**B**) Root Mean Square Deviation (RMSD) of CCHFV-RdRp complexed with all three reported drugs with distinctive colors are highlighted over a period of 10 ns simulations, while, Root Mean Square Fluctuation (RMSF) of all residues during MD simulation are highlighted below. (**C)** Pharmacophoric features projected within the predicted target site of bound ligand with H-bond donors (HBDs) and H-bond acceptors (HBAs) are highlighted in green and red spheres, respectively. Hydrophobic centers are in yellow and exclusion volume spheres are highlighted in grey. (**D)** 2D interaction plot for all three reported drugs complexed with RdRp. (**E)** Per-residue decomposition analysis performed with Amber 16 are presented in bar chart for highly contributing residues of predicted binding site of CCHFV-RdRp.

The structural moieties of binding site of CCHFV-RdRp along with the chemical features of reported compounds (ribavirin, arbidol and T-705) were taken into consideration while creating the guiding pharmacophore model^31,32,34,130^. For this, a 10 ns MD simulation was carried out on docked ligand-RdRp complexes, followed by MMGBSA and per-residue energy decomposition analysis. This method resulted in enhanced pharmacophore modeling based on the highly contributing residues, and thus construction of a concise subset of small compounds for further selection. All three compounds after 10 ns were found in favorable conformations inside the predicted active site configured by all six structurally conserved RdRp motifs **(**Fig. 3A**)**. RMSD plots revealed consistent all-atom backbone stability and all three drugs remained inside the binding pocket throughout 10 ns time period except T-705 (orange trajectory), for which small fluctuations were observed (Fig. 3B). The RMSF for all three complexes agrees with lower fluctuations in binding site residues, especially in the palm subdomain which configured all conserved motifs except motif F (Fig. 3B). The pharmacophoric features of all three compounds are displayed in Fig. 3C. 2D interaction plots of average structures after 10 ns are displayed in Fig. 3D. Per-residues decomposition analysis exhibited similar interaction quantification,calculated with arbidol and T-705 complexed with RdRp respectively. Ser536 (−1.917, −1.86 kcal/mol), Phe534 (−0.636, −0.641 kcal/mol), Glu533 (−1.66. −1.515 kcal/mol), Asp316 (−0.825, −0.863 kcal/mol) and Asn317 (−0.736, −0.874 kcal/mol) were found to be top contributing residues (Fig. 3E). Additionally, Gly315 (−1.542 kcal/mol) and Ser474 (−1.482 kcal/mol) also contributed significantly with T-705. Among these highly contributing residues, strong H-bonds were found between nitrogen backbone atom of Ser536 with arbidol (2.38 Å) and T-705 (2.13 Å) respectively. Apart from this, Asn317 forms a H-bond with arbidol (2.81 Å), while energetically favorable residues, Ser474, Asp475 and Glu533 form H-bonds with T-705 (~3 Å). Other selected residues were involved in hydrophobic interactions **(**Fig. 3C–E**)**.

For RdRp complexed with ribavirin, Ser536 (−3.118 kcal/mol), Ser474 (−1.434 kcal/mol) and Asp475 (−0.637 kcal/mol) are forming H-bonds while other highly contributing residues like Tyr535 (−1.076 kcal/mol), Asn317 (−1.332 kcal/mol) and Leu234 (−0.848 kcal/mol) are mainly involved in hydrophobic interactions (Fig. 3E). Each pharmacophoric feature was used to screen a database with the addition of exclusion volume spheres (as highlighted black) to further narrow the search space for individual compounds.

### Virtual screening workflow to identify putative inhibitors

The library generated after scanning the Mcule database with per-residue energy decomposition based pharmacophoric features were subjected to *in silico* predictions of pharmacokinetics (PK), drug-likeness, toxicity potential, and medicinal chemistry friendliness. This subsequent screening removed substantial hits based as follows: i) Poor ADMET (absorption, distribution, metabolism, excretion, and toxicity) profile as predicted by admetSAR^153^, ORISIS Property Explorer^154^ and PreADMET (https://preadmet.bmdrc.kr/). Many hits failed to show desired ADMET characteristics^155^ due to inhibitory effects on the renal organic cation transporter (ROCT) and to CYP450 enzymes 1A2, 2C9, 2D6, 2C19, and 3A4, together with the prediction to be toxic and carcinogenic. ii) Poor drug-likeness was evaluated by Ghose^156^, Veber (GSK)^157^, Pfizer^141^, Egan (Pharmacia)^158^, Muegge (Bayer)^159^ filters and Abbott bioavailability score^160^. A number of compounds failed to cross these filters devised by established pharmaceutical companies directing the selection of the best molecules for experimental testing.

By these criteria, most virtual hits were found to contain high-risk chemical groups, including epoxides that potentially disturb signal transduction cascades by forming protein adducts^161^ and quinones that lead to severe oxidative stress through the formation of reactive oxygen species (ROS)^162^. This extensive step-wise screening resulted in 337 virtual hits after which each compound was docked into the explicit RdRp binding site as predicted by COACH and 3DLigSite. Subsequently, virtual hits were ranked based on binding affinity estimated by an empirical scoring function of AD Vina^163^. To determine whether the compound is known to be a non-specific aggregator, the compounds were further analyzed using aggregator advisor^164^. Few compounds were found to have a highly similar scaffold (>78%), previously reported as potential aggregators, leaving 17 hits for further analysis (Table S4). Based on the molecular interactions, binding affinities, and ensemble-based docking, the top-ranked three complexes were selected, and docking conformations were visually analyzed (Fig. S3, Detailed ADMET profile is tabulated in Table S5). In order to identify similar chemical scaffolds of these compounds, a 2D similarity-based was performed using SEA (Similarity Ensemble Approach) which relates proteins (from ChEMBL database) based on the set-wise chemical similarities that exceed a certain threshold (Tanimoto similarity coefficient) among their ligands^165^. Based on the results, none of the compounds show structural similarity with any known anti-viral inhibitors. Additionally, ChemMapper also predicted the consensus results, which evaluated the 3D-based similarity^166^. To predict the unfavorable side-effects due to off-target interactions/effects of known molecules and drugs, SwissTargetPrediction web server was used which combines different measures of chemical similarity based on both chemical structure (2D) and molecular shape (3D)^167^. All 3 compounds were found to have less than 0.5 off-target probability based on cross-validation analysis in ChEMBL for human protein ligands (Table S6).

### MD simulations and binding free energy calculations

The RdRp-cmd1, RdRp-cmd2 and RdRp-cmd3, together with RTP, and Fluoro-2′-deoxycytidine (2′-FdC) as two reference compounds, were subjected to 10 ns MD simulations to assess the protein dynamic stability and to analyze decomposed energy contributions of each simulated compound in complex with RdRP. The hydrogen-bond analysis in time, three-dimensional interaction analysis and MSA provided valuable comprehension on the identification of catalytic or inhibitory regions within the CCHFV-RdRp predicted binding site.

### CCHFV-RdRp-cmd1/2/3 complex analysis

**Figure 4:**
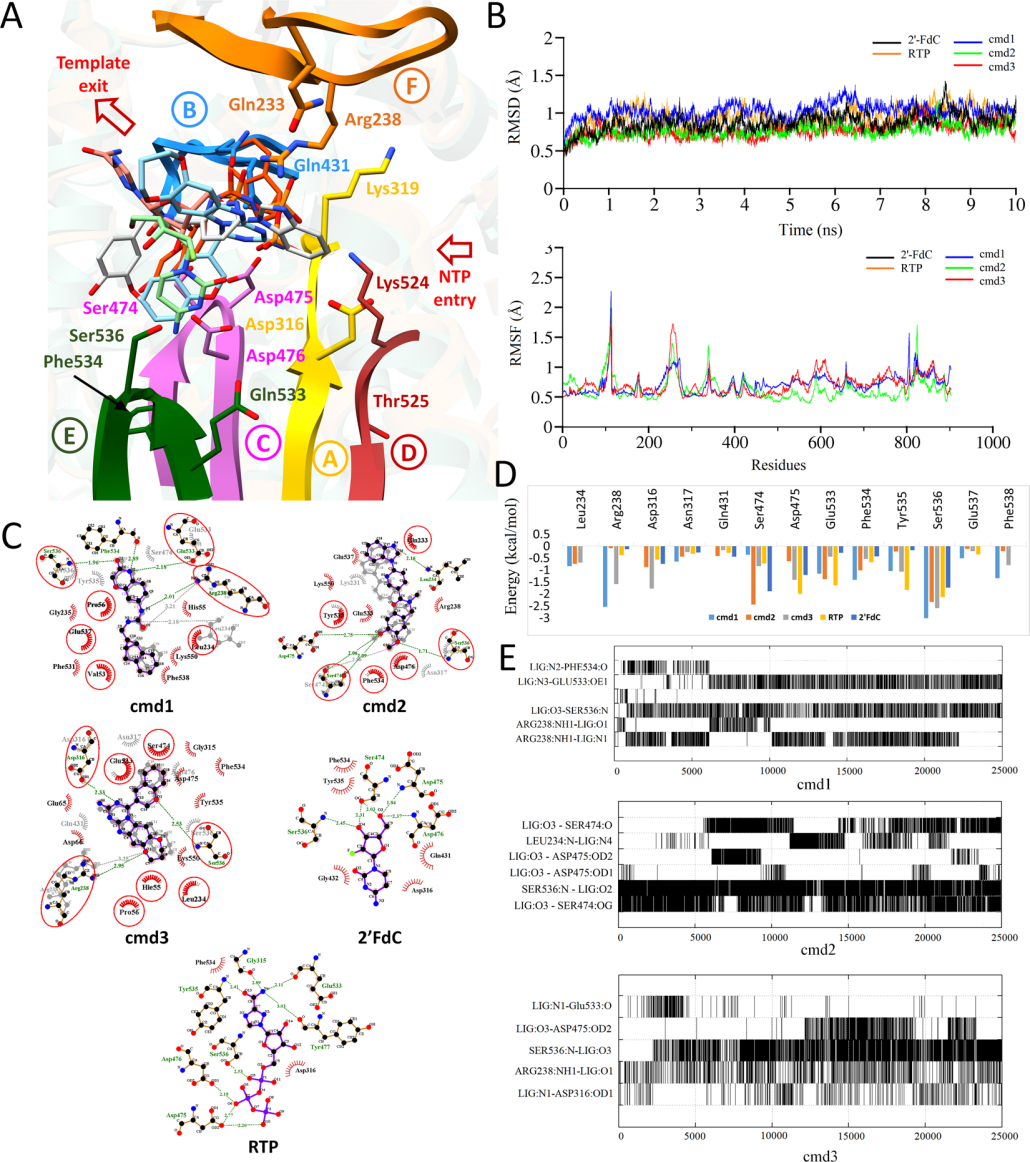
Post-Molecular dynamics (MD) analysis of CCHFV-RdRp complexes. (**A)** MD simulated conformations of cmd1, cmd2, cmd3, and two reference compounds, RTP and 2′FdC in the sticks, inside the predicted binding pocket of CCHFV-RdRp. The arrangement of motifs and colors are same as in Fig. 3A. (**B**) Root Mean Square Deviation (RMSD) of CCHFV-RdRp complexed with presented compounds with distinctive colors are highlighted over a period of 10 ns simulations, while, Root Mean Square Fluctuation (RMSF) of all residues during MD simulation are highlighted below. (**C**) 2D interaction plot is interactively displayed for all presented compounds complexed with RdRp. (**D)** Per-residue decomposition analysis for all 5 compounds performed with Amber 16 is presented in a bar chart for highly contributing residues of the predicted binding site of CCHFV-RdRp. (**E)** H-bond occupancy of particular amino acids in frames throughout 10 ns is displayed for cmd1/2 and 3.

All simulated compounds in complex with CCHFV-RdRp are displayed in Fig. 4A in proximity to all conserved motifs with stable RMSD trajectories converging within a range of 0.5 Å (Fig. 4B). The complexes were further subjected to root-mean-squared-fluctuation (RMSF) analysis which did not indicate regions of extreme fluctuation. Complexes were found to have a stable network of molecular interactions when analyzed through LigPlot (Fig. 4C), per residue decomposition analysis **(**Fig. 4D**)** and H-bond occupancy, defined as the percentage of H-bonds throughout the simulated trajectory **(**Fig. 4E**)**. Total free binding energy shift from 1 ns to 10 ns together with molecular interactions present in docked and simulated conformations tabulated in Table 2.

**Table 2:** Dynamics and energetics of the RdRp complexes with potential hits compared with investigational drugs and two reference compounds RTP and 2′-FdC.

Virtual hits	AD Vina binding affinity (kcal/mol)	RMSD ligand (Å)	Time frame (ns)	MM-GBSA							H-Bond Interaction		
				ΔE_vdw_	ΔE_ele_	ΔE_MM_	ΔG_p_	ΔG_np_	ΔG_sol_	ΔG_tol_	Atom pair	^a^ d_init_ (Å)	^b^ d_MD_ (Å)
cmd1	−8.7	1.472	Initial (1 ns)	−32.62	−24.97	−57.59	45.17	−4.38	40.79	−16.8	LIG:O3-Ser536:N	1.96	nd
			Final (10 ns)	−36.94	−27.59	−64.53	36.67	−5.63	31.04	−33.49	LIG:N3-Glu533:OE1	2.18	nd
			–	–	–	–	–	–	–	–	Arg238:NH1-LIG:N1	nd	2.01
			–	–	–	–	–	–	–	–	Arg238:NH1-LIG:O1	3.21	nd
			–	–	–	–	–	–	–	–	LIG:N2-Phe534:O	nd	2.89
			–	–	–	–	–	–	–	–	LIG:N2-Leu234:O	2.18	nd
cmd2	−8.3	1.719	Initial (1 ns)	−33.41	−19.56	−52.97	39.45	−4.51	34.94	−18.03	LIG:O2-Ser474O	2.86	nd
			Final (10 ns)	−40.72	−31.13	−71.85	46.01	−6.36	39.65	−32.2	LIG:O3-Ser474:OG	nd	2.09
			–	–	–	–	–	–	–	–	LIG:O3-Ser474:O	nd	2.06
			–	–	–	–	–	–	–	–	LIG:O3-Asp475:OD2	nd	2.75
			–	–	–	–	–	–	–	–	Leu234:N-LIG:N4	nd	2.16
			–	–	–	–	–	–	–	–	Ser536:N-LIG:O2	nd	1.71
cmd3	−9.8	1.822	Initial (1 ns)	−34.84	−25.03	−59.87	42.12	−4.33	37.79	−22.08	Ser536:N-LIG:O3	nd	2.53
			Final (10 ns)	−38.91	−37.4	−76.31	45.32	−5.91	39.41	−36.9	Arg238:NH1-LIG:O1	3.25	2.1
			–	–	–	–	–	–	–	–	LIG:N1-Asp316:OD1	3.33	nd
Ribavirin	−6.4	1.32	Initial (1 ns)	−17.99	−56.17	−74.16	66.23	−3.43	62.8	−11.36	Asn317:ND2-LIG:O3	nd	2.31
			Final (10 ns)	−17.72	−49.8	−67.52	58.04	−3.66	54.38	−13.14	Ser536:N-LIG:O4	3.1	nd
			–	–	–	–	–	–	–	–	Ser536:N-LIG:O2	nd	2.74
			–	–	–	–	–	–	–	–	LIG:O5-Asp316:OD2	2.77	nd
			–	–	–	–	–	–	–	–	LIG:O3-Asp316:OD2	2.99	nd
			–	–	–	–	–	–	–	–	LIG:O3-Ser474:OG	1.73	nd
			–	–	–	–	–	–	–	–	LIG:O3-Asp475:OD2	1.55	nd
Favipiravir (T-705)	−6.4	0.841	Initial (1 ns)	−20.4	−17.53	−37.93	31.92	−2.77	29.15	−8.78	LIG:O2-Ser474:OG	nd	2.81
			Final (10 ns)	−17.47	−22.01	−39.48	29.81	−2.71	27.1	−12.38	LIG:O2-Ser474:O	nd	2.92
			–	–	–	–	–	–	–	–	LIG:O2-Asp475:OD2	nd	2.83
			–	–	–	–	–	–	–	–	Ser536:N-LIG:N1	3.11	2.13
			–	–	–	–	–	–	–	–	Ser536:N-LIG:O2	2.84	nd
			–	–	–	–	–	–	–	–	LIG:O2-Phe534:O	2.71	nd
			–	–	–	–	–	–	–	–	LIG:O2-Glu533:OE1	2.96	nd
			–	–	–	–	–	–	–	–	LIG:N3-Glu533:O	nd	2.17
			–	–	–	–	–	–	–	–	LIG:F-Gly432:N	3.03	nd
			–	–	–	–	–	–	–	–	LIG:N3-Gly315:O	3.14	nd
			–	–	–	–	–	–	–	–	LIG:N3-Asp316:OD2	3.16	nd
Arbidol	−7	1.385	Initial (1 ns)	−35.56	−8.35	−43.91	31.19	−4.28	26.91	−17	Ser536:N-LIG:O2	nd	2.83
			Final (10 ns)	−42.64	−11.64	−54.28	36.36	−5.38	30.98	−23.3	Asn317:ND2-LIG:O3	nd	2.81
			–	–	–	–	–	–	–	–	Arg238:NH1-LIG:O1	3.26	nd
Ribavirin triphosphate (RTP)	−6.2	1.435	Initial (1 ns)	−10.9	−25.67	−36.57	31.16	−1.74	29.42	−7.15	LIG:O2-Ser474:OG	nd	2.81
			Final (10 ns)	–20.16	−28.41	−48.58	34.72	−3.17	31.55	−17.02	LIG:O10-Asp475:OD2	3.11	2.20
			–	–	–	–	–	–	–	–	LIG:O6-Asp475:OD2	2.98	2.77
			–	–	–	–	–	–	–	–	LIG:O6-Asp476:OD1	3.11	2.19
			–	–	–	–	–	–	–	–	Ser536:OG-LIG:O3	2.84	2.53
			–	–	–	–	–	–	–	–	LIG:O13-Tyr535:N	nd	2.41
			–	–	–	–	–	–	–	–	LIG:N4-Glu533:O	2.96	2.11
			–	–	–	–	–	–	–	–	LIG:N4-Gly315:O	nd	2.89
			–	–	–	–	–	–	–	–	LIG:N4-Tyr477:O	3.01	3.03
2′FdC	−7.1	1.289	Initial (1 ns)	−17.29	−40.10	−57.39	38.54	−2.91	35.63	−21.76	LIG:OG-Ser474:O3	2.14	2.03
			Final (10 ns)	−19.70	−42.10	−61.8	39.48	−2.35	37.13	−24.67	LIG:OG-Ser474:O4	nd	2.31
			–	–	–	–	–	–	–	–	LIG:N-Ser475:O3	2.27	1.84
			–	–	–	–	–	–	–	–	LIG:O3-Ser476:N	2.70	2.37
			–	–	–	–	–	–	–	–	Ser536:N-LIG:O4	2.01	2.45

^init^Initial distance of H-bond in Angstrom (Å) between atom pair.

^b^Distance calculated after MD simulations for the H-bond between the same atom pair.

### RdRp/cmd1

For RdRp/cmd1, the 2D Ligplot representation shows the importance of residues Arg238, Glu533, Phe534, and Ser536 belong to motif F and E respectively (Fig. 4C). The guanidine sidechain of Arg238 accepted an H-bond from the urea linker. Glu533 and Phe534 accepted an H-bonds from the nitrogen donor in the benzoxazinone moiety which additionally accepted an H-bond from the backbone amide in Ser536. The H-bond trajectory analysis revealed a high occupancy for Arg238, Glu533, and Ser536 with the highest total energy contribution by Ser536 (Fig. 4D,E). Glu537 interacted predominantly electrostatic. However the total interaction energy categorized it as less contributing. Phe534 displayed a lower overall energetic contribution with a high Van-der-Waals (vdw) contributions. This difference could be accounted to solvation effects. Likewise in the generated pharmacophore model, the Ser536 donates an H-bond to the ethyl carboxylate moiety in Arbidol, to the chloropiperazinone moiety in Favipiravir and the ribose moiety in Ribavirin (Fig. 3C), thus representing a common pharmacophoric feature. With a total interaction free energy result of −33.49 kcal/mol after 10 ns simulation and scored similarly in comparison to the other compounds but displays a substantial increase in predicted binding affinity compared to the investigational drugs (Table 2**)**.

### RdRp/cmd2

The 2D Ligplot representation of RdRp/cmd2 reveals the importance of residues belongs to motif F (Leu234), motif D (Ser474, Asp475) and motif E (Ser536) (Fig. 4C). Leu234 donated an H-bond from its backbone amide to the benzimidazole moiety of cmd2. Asp475 accepted an H-bond from the hydroxyl of the α-methoxyfenol moiety. With the same hydroxyl, Ser474 acted as H-bond donor and acceptor resulting in two stable H-bonds. Ser536 donated an H-bond from its backbone amide to the α-methoxy group in the same moiety. The H-bond trajectory analysis disclosed nearly 100 percent occupancy for Ser536. Ser474 showed very high occupancy with its hydroxyl sidechain and fair occupancy with its backbone carbonyl as acceptor. The per-residue decomposition analysis of cmd2 displayed notable contributions with Ser474, Glu533, and Ser536 (Fig. 4D). Glu533, however not present in ligplot, especially mediated electrostatic interactions throughout the simulation. Both Ser474 and Ser536 contributed strongly to the overall interaction energy of −32.2 kcal/mol which ranked notably higher than investigational drugs (Table 2). Moreover, when comparing the residues with the highest energetic contribution seen in RdRp complexed with Ribavirin, Favipiravir and Arbidol (Fig. 3C), cmd2 displayed the most common features and energetically contributing residues.

### RdRp/cmd3

The 2D Ligplot representation of cmd3 displays Arg238, Asp316, and Ser536 as important residues of motif F, A and E interacting through H-bond formation (Fig. 4C). Arg238 donated a H-bond from its guanidine sidechain to the oxygen in the dioxapine moiety of cmd3 and Asp316 accepted a H-bond from the nitrogen in the imidazotriazine moiety. While Ser536 donated a H-bond from its backbone amide to oxygen in the *2H-*chromene moiety. The H-bond analysis showed the importance of residues Arg238, Asp316, Ser536 as significant contributors to the overall free interaction energy (Fig. 4D,E). Arg238 displays notable H-bond occupancy throughout the simulation, with a strong electrostatic contribution. The H-bond with Ser536 was formed during the simulation with prominent occupancy. Glu533 interaction, as labeled important in the structure based pharmacophore analysis, is only minorly contributing to the overall free energy.

Additionally, the H-bond analysis displays a low occupancy. However, the neighboring residue, Glu537, is showing the same pattern of electrostatic interaction as seen in cmd1 (Fig. 4C) and Ribavirin (Fig. 3C). The overall interaction energy was calculated as −36.9 kcal/mol. The three simulated compounds score similarly, while markedly scoring more favorable interaction energy values as compared to the investigational drugs (Table 2).

### RdRp/RTP and 2FdC complexes

Both reference compounds produced overall consensus results compared to top three compounds. The MD simulations shaped the favorable conformation of RTP and 2′FdC inside the predicted binding site (Fig. 4A) and remained stable throughout 10 ns, with no significant fluctuations observed (Fig. 4B). The generated 2D plots of 2′FdC show a conserved network of molecular interactions as seen in three virtual hit compounds (Fig. 4C), where 2′FdC interacted mainly through a network of H-bonds with Ser474, Asp475 and Asp476 in motif D, and Ser536 in motif E **(**Figure D**)**. Similarly, the RTP established a network of H-bonds especially through triphosphates with residues of motif D (Asp475, Asp476), motif E (Glu533, Ser536) and motif A (Gly315) (Fig. 4C). Per-residue energy contributions from interacting residues were also found to be comparable as calculated in virtual hits (Fig. 4D). Evidently, both reference compounds showed expected results in terms of interactions and contributing (Table 2).

## Discussion

Even though CCHF is a very old and well-recognized disease, little effort has been put to eradicate either the disease or its symptoms. Besides ribavirin, T-705 has proven historically reliable in reducing CCHF viremia and has been found to be the most efficacious drug from a cohort of similar agents when used against a variety of CCHFV strains^130,168^. Recently, a screen of candidate nucleoside analog compounds identified 2′-deoxy-2′-fluorocytidine with increased activity in CCHFV compared to ribavirin and T-705^35^ and showed antiviral activity against several unrelated *Bunyaviruses*^36^. Additionally, two repurposed FDA molecules chloroquine and chlorpromazine showed direct activity against CCHFV^39^. However, there are practically no marketed alternatives available so far.

The CCHFV has a complex genome with multiple proteins involved in processes ranging from virus entry into host cells to viral replication and suppression of the host immune system^10,169^. Among these, CCHFV-RdRp is involved in critical mechanisms in the virus life cycle, which involves replication/transcription of vRNA in the cytoplasm of infected cell. Therefore, this target is considered an important target against CCHFV (extensively reviewed by^15,145^). All RdRps for which the crystal structures have been resolved represent a similar right-handed architecture including three subdomains named by their positions in space relative to the palm subdomain^17^, the conserved polymerase motifs (A-F)^145,170^ including the signature motif C (GDD in +sRNA^19^ or SDD in −sRNA viruses)^20^ and the recently discovered motif H^21^. The recently resolved crystal structure of LaCV (L_1750_; residues 1–1750)^21^ revealed the same overall architecture with influenza polymerase^147^ including the NTP addition chamber (active site), entrance and exit tunnels. This despite the low sequence identity. Because of the unavailability of CCHFV-RdRp crystal structure in the protein database, an extensive approach to model CCHFV-RdRp was undertaken to further identify potential anti-CCHFV compounds using integrated computational methods. The *in silico* methods provide a direct and scientifically well-funded basis to guide in *vitro* methods for antiviral drug discovery^70,171^. Computational drug discovery has proven to accelerate the challenging process of designing and optimizing new drug candidates^70,73^. Homology modeling has gained momentum and achieved solid progress with numerous studies reporting refinement of modeled structures followed by MD simulation^92,172–175^.

The final CCHFV-RdRp model was optimized and refined by first selecting the most fitted template, followed by extensive post-MD analysis. Models generated from various structure predictions programs enabled the selection of L_1750_ (PDB ID: 5amq) as the most favorable template, scrutinized by various inclusive structural comparisons (Supplementary information). The final model was generated through MODELLER, by incorporating the spatial secondary structure restraints obtained after 3D structural superimposition of all models with the template (L_1750_). Even with low sequence identity, the generated model revealed a similar overall structure for all RdRp subdomains when compared with L_1750_^17,18^ the identified bridge domain (residues 702–782) and partially the thumb ring (residues 783 to ~951) (Fig. 1A,B). Although certain differences emerged, including the α-ribbon, unique California insertion and priming loop in respective domains (Fig. 1B) but the structural superimposition of CCHFV-RdRp with L_1750_ configured the fingers and thumb subdomains exactly alike insimilar to other polymerases^17,18^ mainly by their positions, relative to palm subdomain (Fig. 1C). Therefore, sequence similarity (especially in the secondary structural elements), suggested structural/functional relationship, possibly conserved motifs corresponding to conserved binding/enzymatic function are a more appropriate measure of query protein relatedness.

Moreover, the CCHFV-RdRp model was formed as determined in L_1750_ and other (−) ssRNA viruses, including motifs A-E in the palm and motif F (premotif A) in finger subdomains^20,21^. Like in L_1750_ and influenza polymerase^147^, the arrangement of core subdomains defined the formation of the active site chamber **(**Fig. 2A**)** which is connected to exterior by four positively charged tunnels **(**Fig. 2C**)**, template entry and exit channels. The overall backbone stability of CCHFV-RdRp (residues 85–951) which was comparable to residues 758–1745 in L_1750_ although the higher stability in L_1750_ was due to the partial modeling of the thumb ring channel in CCHFV-RdRp model which partly defines the template entry^21^ **(**Fig. 1D**)**.

The druggable binding site prediction estimated potential residues which reside inside the conserved polymerase motifs (Fig. 2A**)**. When poliovirus elongation complex structure was superimposed, the conserved Asp residues of signature motif C (Asp475 in model) and A (Asp316 in model) also showed to be in close connection with a divalent cation (Mg^+2^)^176,177^ (Fig. 2A). The importance of aspartate residues in motif C (SDD or GDD) and motif A (Dx_2_KW) is evident from mutational studies which revealed altered polymerase activity in several RdRp viruses^178–184^. Because of the highly conserved similar fold (Table S3), the presence of structural conserved motifs and configuration of uniform spatial arrangement of core RdRp domains suggests a conserved evolutionary link between RNA polymerase viruses^185–187^.

MD-refined and optimized CCHFV-RdRp structure incorporated >90% residues in Ramachandran favored region with 17 outliers (Fig. S2). Therefore, the model seems reliable for elucidating CCHFV inhibitors through a structure-based virtual screening (SBVS) protocol. Molecular dynamics based pharmacophore model generation with reported drugs **(**Fig. 3**)**, ribavirin, arbidol and T-705^31,131,132^, followed by step-wise virtual screening resulted in final hits that were optimized with rigorous post-MD simulations analysis.

For all compounds including RTP and 2′-FdC reference compounds, the molecular dynamics simulation resulted in an energetically favorable interaction, which provided a more clear insight in the binding mode with structurally conserved RdRp motifs in the palm subdomain. The initial docked pose for each compound already revealed a higher binding affinity, in terms of initial AD-Vina Score and overall binding energy calculated by MM/GBSA module in comparison to the investigational drugs (Table 2). Based on pharmacophore model determination using reported drugs **(**Fig. 3**)**, functionally important aspartates of motif A (Asp316) and motif C (Asp475, Asp476), together with Glu533 and Ser536 of motif E were found as major H-bond donor/acceptor in the pharmacophore analysis (Fig. 3C,D), additionally evident from the interaction network in all virtual hit (Fig. 4C). The importance of aspartates in RdRps as reported in CCHFV^184^ and various other mutational studies^178–182^, highly suggesting a conserved binding feature of cmd1, cmd2 and cmd3, along with Glu533 and Ser536. These residues line deep in the predicted binding pocket of RdRp, potentiating it as an anchor for inhibitory molecules.

Cmd2 ranked highest as potential inhibitor, despite slightly lower interaction free energy as compared to the other simulated compounds. Additionally, it showed the highest H-bond occupancy with Ser536 of motif E and Ser474, Asp475 of motif D as compared to the other simulated compounds.

Conclusively, this comprehensive study introduced a multistep computational approach towards the introduction of novel drugs into the area of anti-CCHF therapy. Merging available protocols has proven to be beneficial because of inherent limitations of the individual sub-strategies^70,188^. By combining all presented results, we believe the predicted homology model is reliable to hypothesize the 3D-conformation of RdRp core subdomains with conserved motifs and pharmacophoric features maintained by investigational drugs. The resulting model allowed the identification of potential compounds and revealed a common predicted inhibitory effect which was supported *in silico* with the potent 2′FdC and RTP reference compounds. However, further *in vitro* testing is necessary to evaluate compound efficacy. Moreover, a widely applicable *in silico* drug design strategy is depicted for target enzymes without crystal structure.

## Supplementary information

In-silico structural elucidation of RNA-dependent RNA polymerase towards the identification of potential Crimean-Congo Hemorrhagic Fever Virus inhibitors
