# Supplementary material for: In silico structural elucidation of RNA-dependent RNA polymerase towards the identification of potential Crimean-Congo Hemorrhagic Fever Virus inhibitors
Source: Sci Rep. 2019 May 2;9:6809. doi: 10.1038/s41598-019-43129-2 (PMC6497722; doi:10.1038/s41598-019-43129-2)
Supplement: Supplementary file 1 — In-silico structural elucidation of RNA-dependent RNA polymerase towards the identification of potential Crimean-Congo Hemorrhagic Fever Virus inhibitors [file 41598_2019_43129_MOESM1_ESM.docx]

***In-silico* structural elucidation of RNA-dependent RNA polymerase towards identification of potential Crimean-Congo Hemorrhagic Fever Virus inhibitors**

Muhammad Usman Mirza^1,2^_,_ Michiel Vanmeert^1^, Matheus Froeyen^1*^, Amjad Ali^3^, Shazia Rafique^4^, Muhammad Idrees^4,5^

^1^Department of Pharmaceutical and Pharmacological Sciences, Rega Institute for Medical Research, Medicinal Chemistry, University of Leuven, Leuven B-3000, Belgium

^2^Center for Research in Molecular Medicine (CRiMM), The University of Lahore, Lahore, Pakistan

^3^Department of Genetics, Hazara University, Mansehra, Khyber Pakhtunkhwa, Pakistan

^4^Centre for Applied Molecular Biology (CAMB), University of the Punjab, Lahore, Pakistan

^5^Vice Chancellor Hazara University Mansehra, Pakistan

*Corresponding author

Prof. Matheus Froeyen

^1^Department of Pharmaceutical and Pharmacological Sciences, Rega Institute for Medical Research, Medicinal Chemistry, University of Leuven, Leuven B-3000, Belgium

Email addresses

MUM: [muhammadusman.mirza@kuleuven.be](mailto:muhammadusman.mirza@kuleuven.be)

MV: michiel.vanmeert@[kuleuven.be](http://kuleuven.be)

MF: [mathy.froeyen@kuleuven.be](mailto:mathy.froeyen@kuleuven.be)

AA: amjad.camb@pu.edu.pk

SR: [shaziarafique@gmail.com](mailto:shaziarafique@gmail.com)

MI: idrees.camb@pu.edu.pk

**Molecular dynamics simulation protocol**

The tleap module of AMBER was used to prepare the simulation system, with an octahedral box extending 10.0Å around the solute and periodic box wall with explicit TIP3 water molecules. Prior to energy minimization, charges were neutralized by adding Na + ions. The periodic boundary conditions based on particle mesh Ewald electrostatic method with a cut-off of 10Å for non-bounded interactions were applied. The time step of 1 fs (only for Langevin dynamics during equilibration) or 2 fs along with the SHAKE algorithm was used to constrain the bonds involving hydrogen atoms. Energy minimization was performed using steepest descent minimization of 5000 steps followed by a conjugate gradient minimization of 1000 steps. All atoms of the system were energy minimized with gradually reducing restraint force constant on the protein atoms (from 10 to 0 kcal/mol/Å^2^) to optimize the solvent position. For equilibration, the same stepwise protocol was used started from, i) 10ps heating of system 10K to 300K with a Langevin thermostat (γ = 1.0 ps-1) keeping the volume constant with 5kcal/mol/Å^2^ restraint force constant on protein atom position; ii) same as the first step but 20ps without any positional restraints; iii) 20 ps MD at 300K using Langevin thermostat (γ = 0.5 ps-1) keeping the volume constant without any positional restraints; (iv) initial 50ps MD at 300K using Langevin thermostat (γ = 1.0 ps-1) under constant pressure (1 bar) without any positional restraints; (v) additional 400 ps MD at 300K, constant pressure without positional restraints. A final 100ns simulation was carried out at constant temperature (300 K) and pressure (1 bar). The MD simulation trajectory was analyzed with the CPPTRAJ module of AMBER


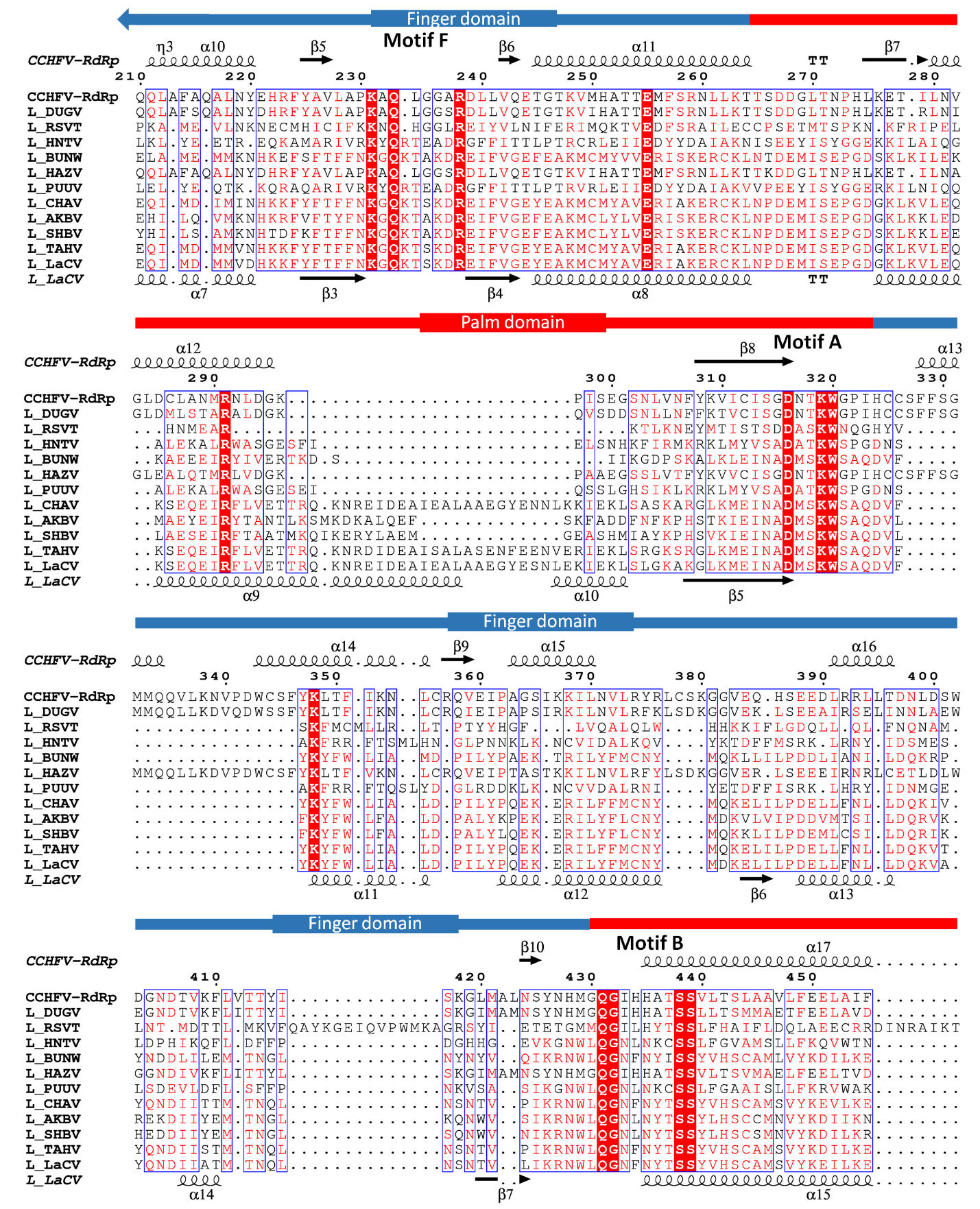


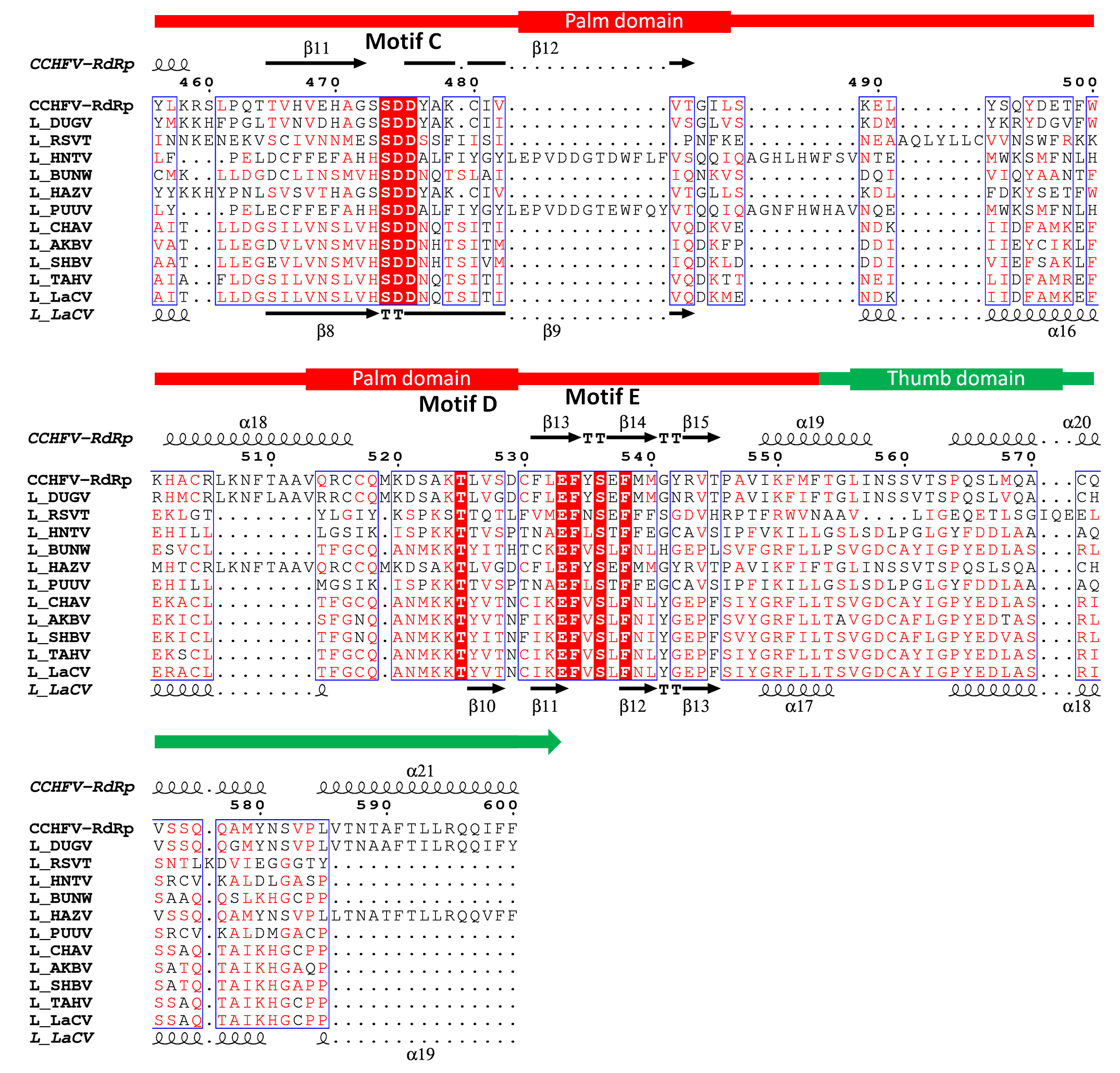


**Figure S1:** Multiple sequence alignment (using T-Coffee) of conserved structural motifs in the central region of Bunyaviridae RdRps. The key residues in these motifs are highlighted red while the similar amino acids are colored red. Alongside, the predicted secondary structural elements forming the active site chamber of CCHFV-RdRp are also mentioned (above the alignment) in comparison with template (LaCV, L_1750_) (below the alignment). The RdRp domains are highlighted as in Figure 1 of main text. Viral RdRps abbreviations and accessions numbers are as follows: Dugbe virus (DUGV), Rice stripe virus (RSVT), Hantaan virus (HNTV), Bunyamwera virus (BUNW), Hazara virus (HAZV), Puumala virus (PUUV), Chatanga virus (CHAV), Akabane virus (AKBV), Schmallenberg virus (SHBV) and Tahyna virus (TAHV).


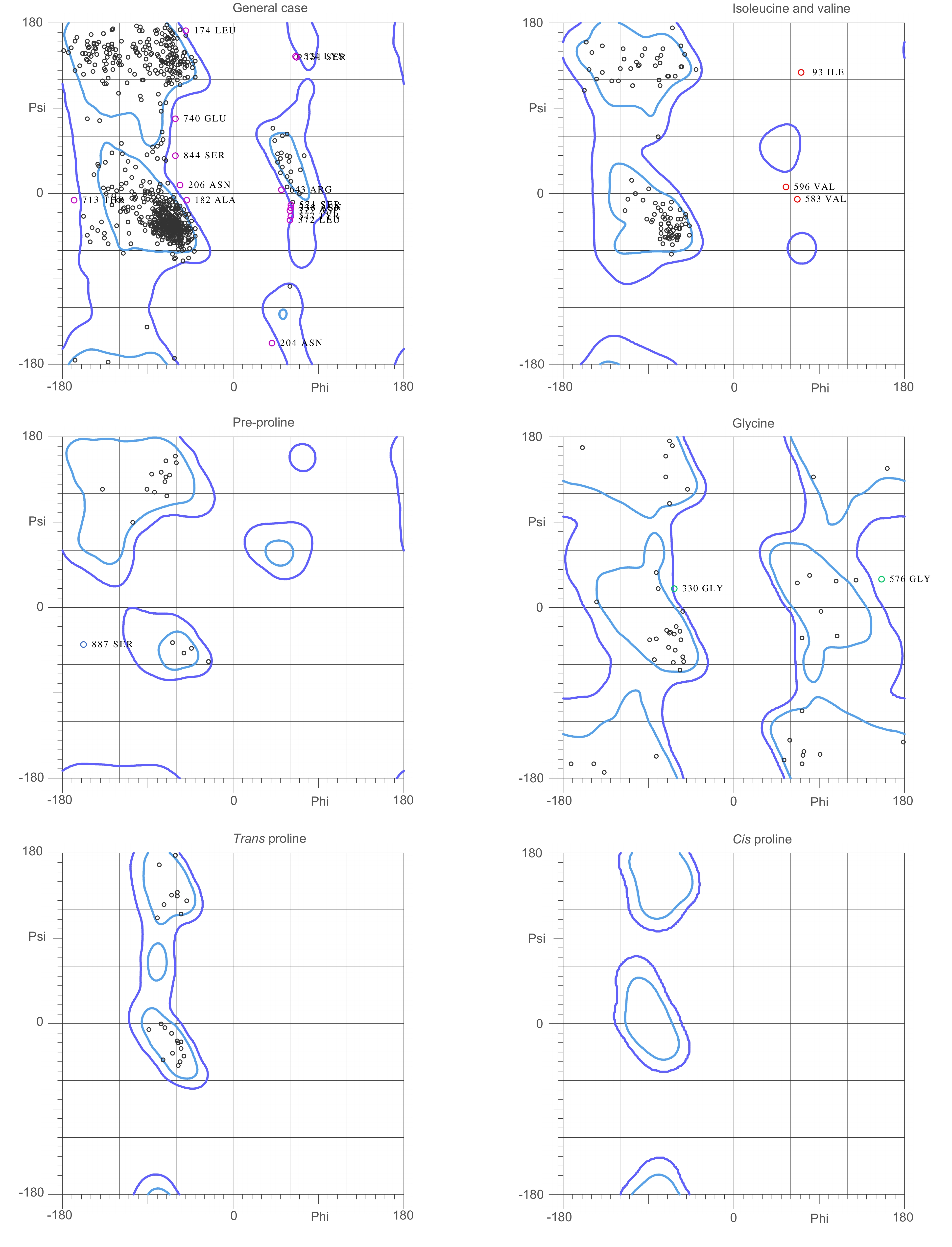


**Figure S2:** Ramachandran plot analysis generated by Molprobity representing 92.12% (876/951) of all residues were in favored (98%) regions. 97.79% (929/951) of all residues were in allowed (>99.8%) regions. There were 17 outliers (phi, psi) as follows: 93 ILE (71.2, 128.9), 131 SER (67.3, 144.9), 174 LEU (-50.9, 172.7), 182 ALA (-49.7, -7.7), 204 ASN (41.9, -158.8), 206 ASN (-56.6, 9.2), 330 GLY (-63.3, 20.8), 338 ASN (61.9, -15.2), 372 LEU (60.7, -28.2), 377 ASP (61.0, -18.9), 524 LYS (66.8, 145.1), 596 VAL (55.4, 7.5), 643 ARG (51.7, 5.0), 713 THR (-168.5, -7.2), 740 GLU (-61.1, 79.0), 844 SER (-61.3, 40.2), 887 SER (-158.6, -39.9).

**
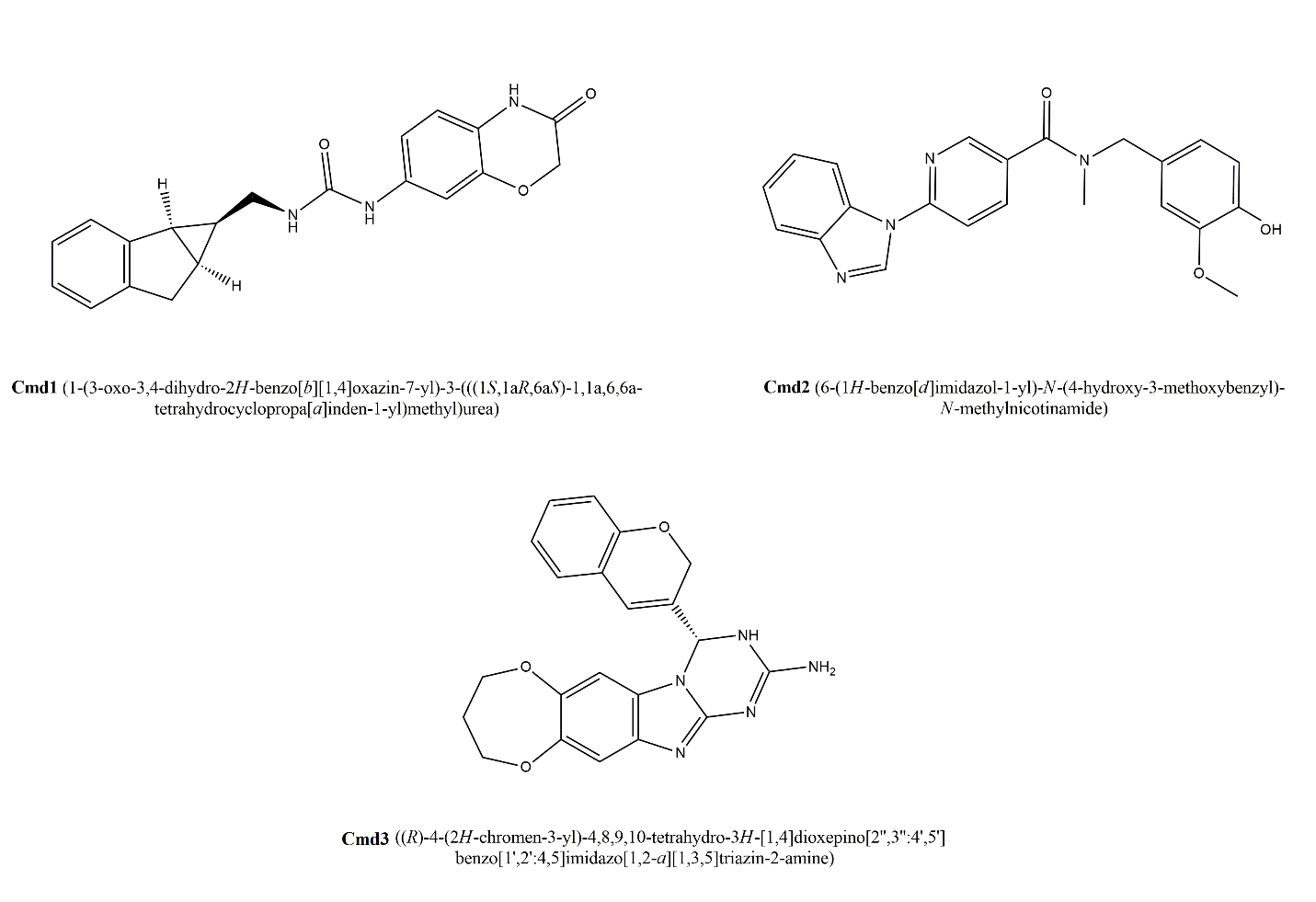
**

**Figure S3: Molecular structures of top hit compounds with IUPAC names.**

**Table S1: Top ranked templates identified by LOMETS meta threading server.**

| **Rank** | **PDB Hit** | **ID1^a^** | **ID2^b^** | **Cov^c^** | **Norm. Zscore^d^** | **Top templates from each threading Program^e^** |
| --- | --- | --- | --- | --- | --- | --- |
|  |  |  |  |  |  |  |
| 1 | 5amq.A | 0.17 | 0.14 | 0.93 | 4.91 | HHpred |
| 2 | 5amq.A | 0.15 | 0.13 | 0.92 | 1.27 | CEthreader |
| 3 | 5amq.A | 0.12 | 0.11 | 0.92 | 1.49 | SparksX |
| 4 | 5twv.B | 0.16 | 0.14 | 0.91 | 1.86 | FFAS3D |
| 5 | 5amq.A | 0.18 | 0.16 | 0.94 | 1.54 | MUSTER |
| 6 | 5vch.A | 0.09 | 0.09 | 0.91 | 2.14 | wMUSTER |
| 7 | 5amq.A | 0.18 | 0.12 | 0.69 | 5.7 | HHsearch |
| 8 | 5amq.A | 0.17 | 0.08 | 0.51 | 3.51 | SP3 |
| 9 | 5amq.A | 0.15 | 0.08 | 0.49 | 1.57 | PPAS |
| 10 | 5ifeC | 0.1 | 0.08 | 0.74 | 2.13 | PROSPECTOR2 |

**Note:**^a^ ID1 is the number of template residues identical to query divided by number of aligned residues.

^b^ ID2 is the number of template residues identical to query divided by query sequence length.

^c^ Cov is equal the number of aligned template residues divided by query sequence length.

^d^ Norm. Zscore is the normalized Z-score of the threading alignments. A normalized Z-score ≥1 means a good alignment.

^e^ Top ranked templates identified by LOMETS different threading programs through deep multiple sequence alignments by iterative sequence homolog searches through multiple sequence databases.

| **Servers used** | **Template results (Query sequence length: 951aa)** | | | | | | | **MATRAS results** | | | | | | **FATCAT results** | | | | |
| --- | --- | --- | --- | --- | --- | --- | --- | --- | --- | --- | --- | --- | --- | --- | --- | --- | --- | --- |
|  | **Template** | **Cov**  **(%)** | **Id**  **(%)** | **Sim**  **(%)** | ***N-*Zscore** | ***P*-value** | **TM** | **N-aa** | **N-sse** | **Sec(%)** | **cRMS** | **SCOP-SF** | **Fold** | **opt-equ** | **opt-RMSD** | **Ch-RMSD** | ***P*-value** | **score** |
| I-TASSER | **5amq.A** | **93** | **19.6** | **38.6** | **4.95** | **n/a** | **0.89** | **761** | **38** | **86.2** | **1.43** | **94.1** | **98.8** | **803** | **1.37** | **1.13** | **0.00E+00** | **1484.68** |
|  | 4wsb.B | 81 | 18.2 | 35 | 4.1 | n/a | 0.634 | 661 | 28 | 79.3 | 3.33 | 55.4 | 90 | 837 | 2.38 | 1.34 | 0.00E+00 | 1813.88 |
|  | 4wrt.B | 83 | 18.4 | 34.1 | 4.7 | n/a | 0.588 | 663 | 30 | 78.3 | 3.44 | 56.9 | 90.6 | 826 | 2.19 | 2.61 | 0.00E+00 | 1729.66 |
|  | 5a22.A | 69 | 14.4 | 30.2 | 1.9 | n/a | 0.503 | 614 | 24 | 81 | 2.23 | 97.7 | 99.1 | 799 | 2.42 | 1.6 | 0.00E+00 | 1768.54 |
|  | 5d98.B | 82 | 17.8 | 33.5 | 3.9 | n/a | 0.552 | 641 | 28 | 74.6 | 3.64 | 48.8 | 84 | 815 | 1.98 | 2.57 | 0.00E+00 | 1784.41 |
| SWISS-MODEL | 5amq.A | 36 | 18.6 | 29 | n/a | n/a | 0.859 | 348 | 22 | 83.9 | 1.44 | 58.3 | 91.3 | 349 | 1.38 | 0.98 | 0.00E+00 | 809.58 |
|  | 5d98.B | 32 | 21.04 | 30 | n/a | n/a | 0.695 | 310 | 22 | 87.7 | 1.44 | 64.8 | 94.1 | 292 | 3.03 | 3.73 | 8.12E-07 | 416.46 |
| LOMETS | **5amq.A** | **93** | **19.2** | **39.8** | **5.07** | **n/a** | **0.808** | **729** | **31** | **77.5** | **2.58** | **45.9** | **91.3** | **639** | **2.47** | **3.56** | **0.00E+00** | **912** |
|  | 4wsb.B | 83 | 17.2 | 35.2 | 4.06 | n/a | 0.611 | 608 | 24 | 67.3 | 4.36 | 43.5 | 79.2 | 612 | 3.2 | 4.89 | 9.32E-08 | 1014.24 |
|  | 4wrt.B | 85 | 17.6 | 34.4 | 4.73 | n/a | 0.602 | 575 | 24 | 68.8 | 5.81 | 42.3 | 78.1 | 618 | 3.18 | 4.12 | 1.17E-06 | 960 |
|  | 5d98.B | 85 | 17.4 | 34 | 4.19 | n/a | 0.671 | 508 | 22 | 70.8 | 6.95 | 39.7 | 68.9 | 642 | 4.09 | 5.89 | 2.50E-04 | 865.29 |
| RAPTORX | **5amq.A** | **100** | **18.2** | **38.4** | **n/a** | **8.50E-11** | **0.809** | **802** | **43** | **88.2** | **4.9** | **97.5** | **99.1** | **854** | **1.68** | **8.95** | **1.24E-06** | **1625.03** |
| MUSTER | **5amq.A** | **94** | **18.6** | **37.4** | **1.39** | **n/a** | **0.821** | **734** | **36** | **85.2** | **3.96** | **55.6** | **91.1** | **647** | **4.69** | **11.24** | **4.20E-07** | **1205.77** |
|  | 4wsb.B | 89 | 16.8 | 36.4 | 1.13 | n/a | 0.552 | 754 | 36 | 84.5 | 3.08 | 97.5 | 99.1 | 601 | 3.46 | 11.32 | 1.76E-04 | 699.85 |
|  | 4wrt.B | 87 | 17 | 34.8 | 1.11 | n/a | 0.626 | 647 | 37 | 87.9 | 2.81 | 98.3 | 99 | 565 | 3.33 | 14.96 | 6.79E-05 | 774.29 |
| **^a^**MODELLER | **5amq.A** | **100** | **18.2** | **38.8** | **n/a** | **n/a** | **0.915** | **834** | **48** | **88.5** | **1.45** | **97.5** | **99.1** | **901** | **1.21** | **1.55** | **0.00E+00** | **2401.57** |
|  | 4wsb.B | 100 | 15.4 | 36.6 | n/a | n/a | 0.657 | 686 | 32 | 84.8 | 3.13 | 97.6 | 99.1 | 785 | 3.08 | 5.47 | 0.00E+00 | 1910 |
|  | 5d98.B | 100 | 16.8 | 36 | n/a | n/a | 0.525 | 662 | 30 | 76.5 | 6.28 | 43.7 | 79.3 | 616 | 3.04 | 6.41 | 1.38E-09 | 1254.03 |

**Table S2: Templates comparisons to identify the template reliability through structural similarities and differences**

**Note:** Cov, a percentage of aligned template residues divided by query sequence length; ID, a percentage of template residues identical to query divided by number of aligned residues. TM, TM-align that perform accurate structural alignment for sequence independent structure comparisons (a score > 0.5 assume highly similar fold in SCOP/CATH); N-aa, Number of compared (aligned) residues; N-sse, Number of compared (aligned) secondary structural elements; cRMS : Root mean square deviation (angstrom) of Calpha atom positions of aligned residues, after optimal superimposition. SCOP-SF/Fold, a reliability factor which represent the probability that a structure pair is classified as the same Superfamily/Fold relationship of SCOP database. opt-equ, Number of equivalent residues in the alignment; opt-RMSD; root mean square deviation (RMSD) of aligned Cα atoms of the input structures, with one input structure rearranged if flexibility is detected (i.e., twists are introduced in the alignment), ch-RMSD, rmsd of aligned Cα atoms of the input structures, without structural rearrangement; P-value, a FATCAT score to evaluate the significance of structural similarity detected by FATCAT (Structure pairs with probability < 0.05 are significantly similar). The FACTACT similarity score incorporates the FATCAT chaining score, RMSD of the resulting superposition, the number of equivalent positions in the alignment and the number of twists.

^a^ Final model was built exclusively through a restrained-based approach in MODELLER.v9.1 using 5amq.A as most fitted-template based on conserved structural attributes obtained from multiple 3D structural alignments of all models (built from other programs) with 5amq.A. The criteria included the query length, similarity, normalized Z-score, and assumption of having significant similar fold based on TM-align (score), FATCAT (P-value) and MATRAS program attributes, which evidently represent the comparable aligned SSEs and structural similarity having lowest RMSD without twists. The most-fitted template was used along with the secondary structural information obtained by manual curation after superimposition between all generated models (in step 2) and template. The extracted spatial secondary structure restraints were implemented to model the final structure using secondary structure module of MODELLER after adjusting target-template alignment to reduce the number of misaligned residues.

**Table S3: Reverse template comparison vs structures in PDB using ProFunc reverse template search program**

| **Hit no.** | **Matched PDB entry** | **E-value** | **RMSD (Å)** | **Similarity score** | **Sequence align.attributes** | | **Structural attributes** | | | |
| --- | --- | --- | --- | --- | --- | --- | --- | --- | --- | --- |
|  |  |  |  |  | **Seq. lengths query/target** | **Seq id (%)** | **Neighbours id/sim** | **No. of equivalenced residues** | **Longest fitted segment** | **Structural similarity (%)** |
| 1 | 5amq | 4.48E-09 | 0.94 | 351.62 | 951 / 742 | 17.92 | 17 / 10 . | 39 | 154 / 359 | 87.9 |
| 2 | 4wrt | 1.77E-08 | 0.83 | 335.6 | 951 / 745 | 20.94 | 16 / 10 . | 36 | 90 / 293 | 81.8 |

**Note:** ^a^ Hit number identifies the best ranked hit from PDB (against a representative subset of structures in the PDB).

^b^ The value that represent the significance of hit i.e. E-value <1.00 E-0.6 represents certain matches.

^c^ root-mean-square-deviation between Cα between template and query structures.

^d^ gives the similarity between the neighborhood around the matched side chains in the query structure and the neighborhood around the template side chains in their parent structure.

^e^ represents the sequence lengths between query and template and corresponding percentage identity.

^f^ the first two measures the number of structurally equivalent residues, lying within 10Å of the template center in both structure (template and query), which are of identical and similar amino acid types, respectively. The higher the numbers, particularly the first, the more similar the template environments in both structures. Where the first number is greater than about 16 the similarity starts to take on a real significance, and this is encapsulated in the similarity score. The second two measures how structurally significant superimposition between query and template structures. The criteria of obtaining the values from structural alignment as follows, a sliding window of seven residues lengthwise is slide along the alignment and the C-alpha atoms of the equivalenced residues in both structures are superposed. If the fit gives an rmsd below the cut-off of 3.0Å the length of the window size is extended and the superposition performed again. And so on, until extending the window further gives an rmsd above the cut-off value. The segments are ranked based on longest fitted ones on top. The overall structural similarity indicates the percentage of equivalenced residue-pairs by alignment that lie in one or more fitted segments.

**Table S4: Top 17 hits after step-wise filtering in virtual screening pipeline**

| Molecular Structures | Drug Likeness filters | | Molecular Structures | Drug Likeness filters | |
| --- | --- | --- | --- | --- | --- |
| \| 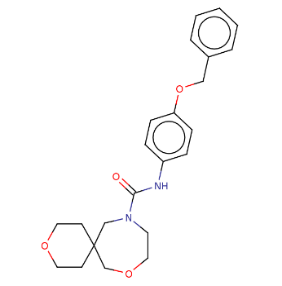  N-[4-(benzyloxy)phenyl]-3,8-dioxa-11-azaspiro[5.6]dodecane-11-carboxamide \| \| --- \| \| | Veber, Egan, GSK 4/400 | ✓ | \| 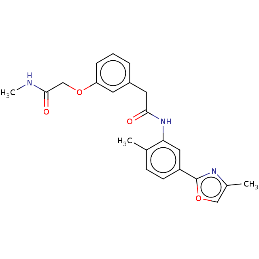  N-methyl-2-[3-({[2-methyl-5-(4-methyl-1,3-oxazol-2- yl)phenyl]carbamoyl}methyl)phenoxy]acetamide \| \| --- \| \| | Veber, Egan, GSK 4/400 | ✓ |
|  | Consensus LogP | 3.03 |  | Consensus LogP | 2.93 |
|  | PAINS, Brenk filter | ✓ |  | PAINS, Brenk filter | ✓ |
|  | Aggregator Advisor | ✓ |  | Aggregator Advisor | ✓ |
|  | Muegge | ✓ |  | Muegge | ✓ |
|  | QpplogHER | -4.59 |  | QpplogHER | -6.27 |
|  | QppCaco | 3731.5 |  | QppCaco | 424.15 |
|  | QppMDCK | 2951.8 |  | QppMDCK | 363.4 |
|  | Docking Score (Kcal/mol) | -8.6 |  | Docking Score (Kcal/mol) | -8.6 |
| \| 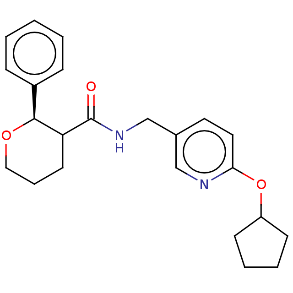  (2R,3S)-N-{[6- (cyclopentyloxy)pyridin-3-yl]methyl}-2-phenyloxane-3-carboxamide \| \| --- \| \| | Veber, Egan, GSK 4/400 | ✓ | \| 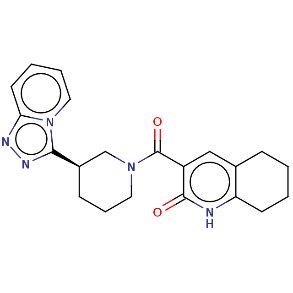  3-[(3R)-3-{[1,2,4]triazolo[4,3-a]pyridin-3-yl}piperidine-1-carbonyl]-1,2,5,6,7,8-hexahydroquinolin-2-one \| \| --- \| \| | Veber, Egan, GSK 4/400 | ✓ |
|  | Consensus LogP | 3.23 |  | Consensus LogP | 2.28 |
|  | PAINS, Brenk filter | ✓ |  | PAINS, Brenk filter | ✓ |
|  | Aggregator Advisor | ✓ |  | Aggregator Advisor | ✓ |
|  | Muegge | ✓ |  | Muegge | ✓ |
|  | QpplogHER | -4.58 |  | QpplogHER | -5.16 |
|  | QppCaco | 3620.1 |  | QppCaco | 361.3 |
|  | QppMDCK | 2924.9 |  | QppMDCK | 164.6 |
|  | Docking Score (Kcal/mol) | -8.5 |  | Docking Score (Kcal/mol) | -8.5 |
| \| 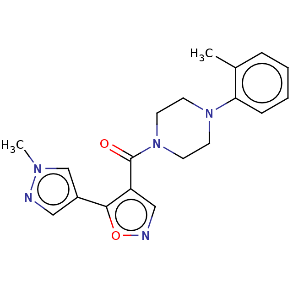  1-[5-(1-methyl-1H-pyrazol-4-yl)-1,2-oxazole-4-carbonyl]-4-(2-methylphenyl)piperazine \| \| --- \| \| | Veber, Egan, GSK 4/400 | ✓ | 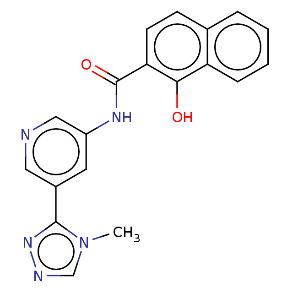   \| 1-hydroxy-N-[5-(4-methyl-4H-1,2,4-triazol-3-yl)pyridin-3-yl]naphthalene-2-carboxamide \| \| --- \| \| | Veber, Egan, GSK 4/400 | ✓ |
|  | Consensus LogP | 1.84 |  | Consensus LogP | 2.08 |
|  | PAINS, Brenk filter | ✓ |  | PAINS, Brenk filter | ✓ |
|  | Aggregator Advisor | ✓ |  | Aggregator Advisor | ✓ |
|  | Muegge | ✓ |  | Muegge | ✓ |
|  | QpplogHER | -4.73 |  | QpplogHER | -6.31 |
|  | QppCaco | 741.39 |  | QppCaco | 348.5 |
|  | QppMDCK | 358.05 |  | QppMDCK | 158.3 |
|  | Docking Score (Kcal/mol) | -8.4 |  | Docking Score (Kcal/mol) | -8.3 |
| \| 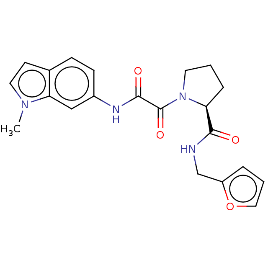  (2S)-N-[(furan-2-yl)methyl]-1-{[(1-methyl-1H-indol-6-yl)carbamoyl]carbonyl}pyrrolidine-2-carboxamide \| \| --- \| \| | Veber, Egan, GSK 4/400 | ✓ | 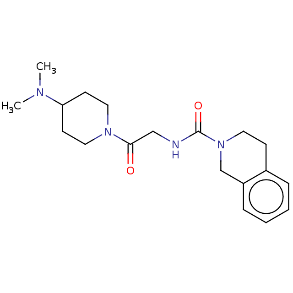   \| N-{2-[4-(dimethylamino)piperidin-1-yl]-2-oxoethyl}-1,2,3,4-tetrahydroisoquinoline-2-carboxamide \| \| --- \| \| | Veber, Egan, GSK 4/400 | ✓ |
|  | Consensus LogP | 1.32 |  | Consensus LogP | 1.45 |
|  | PAINS, Brenk filter | ✓ |  | PAINS, Brenk filter | ✓ |
|  | Aggregator Advisor | ✓ |  | Aggregator Advisor | ✓ |
|  | Muegge | ✓ |  | Muegge | ✓ |
|  | QpplogHER | -4.17 |  | QpplogHER | -3.26 |
|  | QppCaco | 932.6 |  | QppCaco | 176.51 |
|  | QppMDCK | 672.8 |  | QppMDCK | 220.8 |
|  | Docking Score (Kcal/mol) | -8.2 |  | Docking Score (Kcal/mol) | -8.2 |
| \| 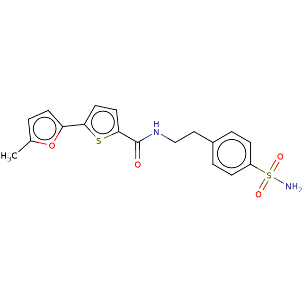  5-(5-methylfuran-2-yl)-N-[2-(4-sulfamoylphenyl)ethyl]thiophene-2-carboxamide \| \| --- \| \| | Veber, Egan, GSK 4/400 | ✓ | 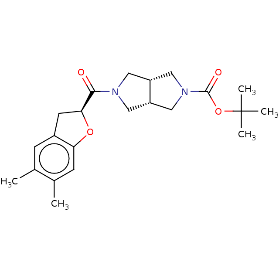   \| tert-butyl (3aR,6aS)-5-[(2S)-5,6-dimethyl-2,3-dihydro-1-benzofuran-2-carbonyl]-octahydropyrrolo[3,4-c]pyrrole-2-carboxylate \| \| --- \| \| | Veber, Egan, GSK 4/400 | ✓ |
|  | Consensus LogP | 2.75 |  | Consensus LogP | 2.93 |
|  | PAINS, Brenk filter | ✓ |  | PAINS, Brenk filter | ✓ |
|  | Aggregator Advisor | ✓ |  | Aggregator Advisor | ✓ |
|  | Muegge | ✓ |  | Muegge | ✓ |
|  | QpplogHER | -6.11 |  | QpplogHER | -4.09 |
|  | QppCaco | 264.62 |  | QppCaco | 1239.5 |
|  | QppMDCK | 167.64 |  | QppMDCK | 1070.47 |
|  | Docking Score (Kcal/mol) | -8.1 |  | Docking Score (Kcal/mol) | -8.1 |
| \| 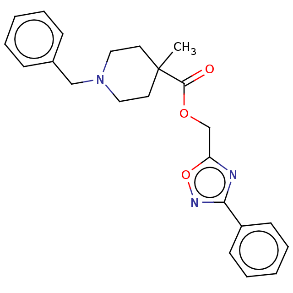  (3-phenyl-1,2,4-oxadiazol-5-yl)methyl 1-benzyl-4-methylpiperidine-4-carboxylate \| \| --- \| \| | Veber, Egan, GSK 4/400 | ✓ | 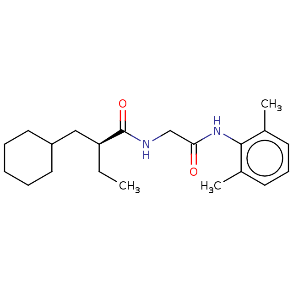   \| (2S)-2-(cyclohexylmethyl)-N-{[(2,6-dimethylphenyl)carbamoyl]methyl}butanamide \| \| --- \| \| | Veber, Egan, GSK 4/400 | ✓ |
|  | Consensus LogP | 3.73 |  | Consensus LogP | 3.17 |
|  | PAINS, Brenk filter | ✓ |  | PAINS, Brenk filter | ✓ |
|  | Aggregator Advisor | ✓ |  | Aggregator Advisor | ✓ |
|  | Muegge | ✓ |  | Muegge | ✓ |
|  | QpplogHER | -7.8 |  | QpplogHER | -3.65 |
|  | QppCaco | 320.18 |  | QppCaco | 2896.8 |
|  | QppMDCK | 159.81 |  | QppMDCK | 2343.4 |
|  | Docking Score (Kcal/mol) | -8.1 |  | Docking Score (Kcal/mol) | -8 |
|  | | | | | |
| \| 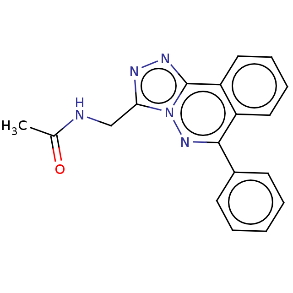  N-({6-phenyl-[1,2,4]triazolo[3,4-a]phthalazin-3-yl}methyl)acetamide \| \| --- \| \| | Veber, Egan, GSK 4/400 | ✓ | \| 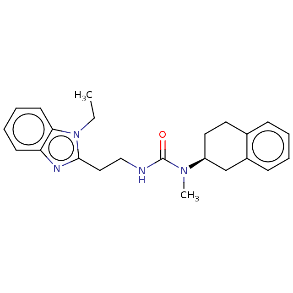  3-[2-(1-ethyl-1H-1,3-benzodiazol-2-yl)ethyl]-1-methyl-1-[(2S)-1,2,3,4-tetrahydronaphthalen-2-yl]urea \| \| --- \| \| | Veber, Egan, GSK 4/400 | ✓ |
|  | Consensus LogP | 2.36 |  | Consensus LogP | 3.19 |
|  | PAINS, Brenk filter | ✓ |  | PAINS, Brenk filter | ✓ |
|  | Aggregator Advisor | ✓ |  | Aggregator Advisor | ✓ |
|  | Muegge | ✓ |  | Muegge | ✓ |
|  | QpplogHER | -4.88 |  | QpplogHER | -5.16 |
|  | QppCaco | 550.6 |  | QppCaco | 1610.04 |
|  | QppMDCK | 422.6 |  | QppMDCK | 1370.9 |
|  | Docking Score (Kcal/mol) | -8 |  | Docking Score (Kcal/mol) | -8 |
| 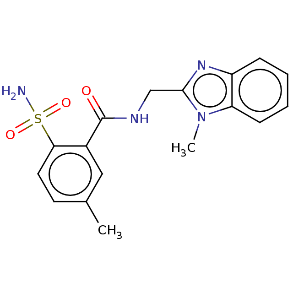   \| 5-methyl-N-[(1-methyl-1H-1,3-benzodiazol-2-yl)methyl]-2-sulfamoylbenzamide \| \| --- \| \| | Veber, Egan, GSK 4/400 | ✓ | \| 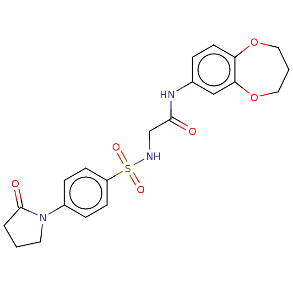  N-(3,4-dihydro-2H-1,5-benzodioxepin-7-yl)-2-[4-(2-oxopyrrolidin-1-yl)benzenesulfonamido]acetamide \| \| --- \| \| | Veber, Egan, GSK 4/400 | ✓ |
|  | Consensus LogP | 1.33 |  | Consensus LogP | 1.74 |
|  | PAINS, Brenk filter | ✓ |  | PAINS, Brenk filter | ✓ |
|  | Aggregator Advisor | ✓ |  | Aggregator Advisor | ✓ |
|  | Muegge | ✓ |  | Muegge | ✓ |
|  | QpplogHER | -5.42 |  | QpplogHER | -6.13 |
|  | QppCaco | 356.1 |  | QppCaco | 339.91 |
|  | QppMDCK | 164.5 |  | QppMDCK | 154.10 |
|  | Docking Score (Kcal/mol) | -7.9 |  | Docking Score (Kcal/mol) | -7.9 |
| \| 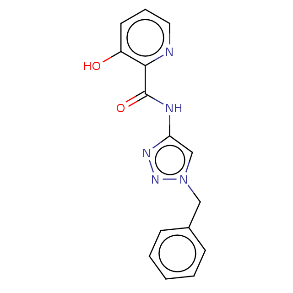  N-(1-benzyl-1H-1,2,3-triazol-4-yl)-3-hydroxypyridine-2-carboxamide \| \| --- \| \| | Veber, Egan, GSK 4/400 | ✓ |  | | |
|  | Consensus LogP | 1.28 |  |  |  |
|  | PAINS, Brenk filter | ✓ |  |  |  |
|  | Aggregator Advisor | ✓ |  |  |  |
|  | Muegge | ✓ |  |  |  |
|  | QpplogHER | -5.504 |  |  |  |
|  | QppCaco | 365.9 |  |  |  |
|  | QppMDCK | 333.58 |  |  |  |
|  | Docking Score (Kcal/mol) | -7.8 |  |  |  |

Veber, Egan, GSK 4/400 (Pfizer) are already explained in the main text; Consensus LogP: Average predictions for all five widely used lipophilicity estimation methods which includes, iLOGP, XLOGP3, WLOGP, MLOGP, and SILICOS-IT; QpplogHER: Predicted IC50 value for blockage of HERG K+ channels (concern below -5); QppCaco: Predicted apparent Caco-2 cell permeability in nm/sec (concerns <25 poor, >500 great).; QppMDCK: Predicted apparent MDCK cell permeability in nm/sec (concerns <25 poor, >500 great)

**Table S5: Detailed Pharmacokinetics and ADMET profile of top 3 hits**

| **Attributes** | **cmd1** | **cmd2** | **cmd3** |
| --- | --- | --- | --- |
| **Molecular Property** | | | |
| Mass | 349.3823 | 388.4183 | 389.4066 |
| logP | 3.3268 | 3.4069 | 3.1863 |
| H-bond acceptors | 6 | 7 | 8 |
| H-bond donors | 3 | 1 | 2 |
| Rotatable bonds | 5 | 6 | 1 |
| TPSA (Total Polar solvent area) | 79.46 | 80.48 | 95.92 |
| Refractivity | 100.4101 | 109.7645 | 115.3141 |
| Atoms | 45 | 49 | 48 |
| Rings | 5 | 4 | 6 |
| Heavy atoms | 26 | 29 | 29 |
| Hydrogen atoms | 19 | 20 | 19 |
| Heteroatoms | 6 | 7 | 8 |
| N/O atoms | 6 | 7 | 8 |
| Inorganic atoms | 0 | 0 | 0 |
| Halogen atoms | 0 | 0 | 0 |
| Chiral centers | 3 | 0 | 1 |
| R/S chiral centers | 3 | 0 | 1 |
| Unknown chiral centers | 0 | 0 | 0 |
| Undefined chiral centers | 0 | 0 | 0 |
| Stereo double bonds | 0 | 0 | 0 |
| Cis/trans stereo double bonds | 0 | 0 | 0 |
| Unknown stereo double bonds | 0 | 0 | 0 |
| Undefined stereo double bonds | 0 | 0 | 0 |
| **Water Solubility** | | | |
| Water solubility | -3.63 | -3.02 | -3.31 |
| Solubility class | Soluble | Moderately Soluble | Soluble |
| **Druglikeness** | | | |
| Lipinski | Yes; 0 violation | Yes; 0 violation | Yes; 0 violation |
| Ghose filter | Yes | Yes | Yes |
| Veber (GSK) filter | Yes | Yes | Yes |
| Egan (Pharmacia) filter | Yes | Yes | Yes |
| Muegge (Bayer) filter | Yes | Yes | Yes |
| Abbott Bioavailability score | 0.55 | 0.55 | 0.55 |
| Druglikeness score | 0.966 | 0.758 | 0.966 |
| **Medicinal Chemistry (Friendly)** | | | |
| PAINS | 0 alert | 0 alert | 0 alert |
| Brenk structural alert | 0 alert | 0 alert | 0 alert |
|  | | | |
| **ADMET** | | | |
| **A (Absorption)** | | | |
| Human Intestinal Absorption (HIA) | + | + | + |
| Human oral bioavailability (HOB) | + | + | - |
| Caco-2 permeability | - | - | - |
|  | | | |
| **D (Distribution)** | | | |
| Plasma protein binding | 0.968 | 0.853 | 0.989 |
| P-glycoprotein inhibitior | - | + | - |
| P-glycoprotein substrate | - | - | + |
| Blood Brain Barrier | + | + | + |
|  | | | |
| **M (Metabolism)** | | | |
| *CYP Inhibitors and Substrates* | | | |
| CYP1A2 inhibition | - | - | + |
| CYP2C19 inhibition | + | - | - |
| CYP2C9 inhibition | - | + | - |
| CYP2C9 substrate | - | - | - |
| CYP2D6 inhibition | - | - | - |
| CYP2D6 substrate | - | - | - |
| CYP3A4 inhibition | - | - | + |
| CYP3A4 substrate | + | + | + |
|  | | | |
| **E (Excretion)** | | | |
| Renal Organic Cation Transporter | - | - | - |
|  | | | |
| **T (Toxicity)** | | | |
| Carcinogenicity | no | no | no |
| Mutagenic | no | no | no |
| Irritant | no | no | no |
|  | | | |
| **QikProp Permeability assessment** | | | |
| QpplogHER | -5.243 | -6.377 | -5.111 |
| QppCaco | 408.44 | 631.63 | 610.33 |
| QppMDCK | 230.28 | 301.07 | 290.11 |

ADMET, absorption distribution metabolism excretion and toxicity; BBB, blood–brain barrier; HIA, human intestinal absorption; CYP450, cytochrome P450; Veber Rule, Bad or Good oral bioavailability rule (rotatable bonds ≤ 10) and (TPSA ≤ 140 Å or H-Bonds Donors + H-Bonds Acceptors ≤ 12); Egan Rule, Bad or Good oral bioavailability rule (0 ≥ TPSA ≤ 132) and (−1 ≥ logp ≤ 6).

**Table S6: Prediction of off-target identification using SwissTargetPrediction**

| **Cmds** | **Target** | **Uniprot ID** | **Gene Code** | **ChEMBL ID** | **Probability^a^** | **No. of similar**  **cmpds (3D)** | **No. of similar cmpds (2D)** | **Target Class** |
| --- | --- | --- | --- | --- | --- | --- | --- | --- |
| **cmd1** | Microtubule-associated protein tau | P10636 | MAPT | CHEMBL1293224 | 0.42 | 1531 | 19 | Unclassified |
|  | Complex | Q05586&Q13224 | GRIN1 | CHEMBL1907603 | 0.39 | 24 | 1 | Ion channel |
|  | Adenosine receptor A2a | P29274 | ADORA2A | CHEMBL251 | 0.35 | 236 | 1 | Membrane receptor |
|  | Adenosine receptor A2b | P29275 | ADORA2B | CHEMBL255 | 0.35 | 192 | 1 | Membrane receptor |
|  | Adenosine receptor A1 | P30542 | ADORA1 | CHEMBL226 | 0.35 | 276 | 1 | Membrane receptor |
|  | Melatonin receptor type 1A | P48039 | MTNR1A | CHEMBL1945 | 0.35 | 329 | 1 | Membrane receptor |
|  | Melatonin receptor type 1B | P49286 | MTNR1B | CHEMBL1946 | 0.35 | 329 | 1 | Membrane receptor |
|  | Melatonin-related receptor | Q13585 | GPR50 | n/a | 0.35 | 329 | 1 | Membrane receptor |
|  | Transient receptor potential cation channel subfamily V member 1 | Q8NER1 | TRPV1 | CHEMBL4794 | 0.33 | 173 | 37 | Ion channel |
|  | Transient receptor potential cation channel subfamily V member 4 | Q9HBA0 | TRPV4 | CHEMBL3119 | 0.33 | 118 | 37 | Ion channel |
|  | Transient receptor potential cation channel subfamily V member 2 | Q9Y5S1 | TRPV2 | CHEMBL5051 | 0.33 | 118 | 37 | Ion channel |
|  | 5-hydroxytryptamine receptor 2A | P28223 | HTR2A | CHEMBL224 | 0.33 | 222 | 22 | Membrane receptor |
|  | Glucocorticoid receptor | P04150 | NR3C1 | CHEMBL2034 | 0.33 | 163 | 4 | Transcription Factor |
|  | Mineralocorticoid receptor | P08235 | NR3C2 | CHEMBL1994 | 0.33 | 171 | 5 | Transcription Factor |
|  | D(2) dopamine receptor | P14416 | DRD2 | CHEMBL217 | 0.31 | 608 | 67 | Membrane receptor |
| **cmd2** | Microtubule-associated protein tau | P10636 | MAPT | CHEMBL1293224 | 0.38 | 1829 | 56 | Unclassified |
|  | Poly [ADP-ribose] polymerase 1 | P09874 | PARP1 | CHEMBL3105 | 0.31 | 374 | 61 | Enzyme |
|  | Adenosine receptor A2a | P29274 | ADORA2A | CHEMBL251 | 0.29 | 242 | 50 | Membrane receptor |
|  | Adenosine receptor A2b | P29275 | ADORA2B | CHEMBL255 | 0.29 | 230 | 47 | Membrane receptor |
|  | Adenosine receptor A1 | P30542 | ADORA1 | CHEMBL226 | 0.29 | 290 | 52 | Membrane receptor |
|  | Muscleblind-like protein 1 | Q9NR56 | MBNL1 | CHEMBL1293317 | 0.28 | 745 | 73 | Unclassified |
|  | Muscleblind-like protein 2 | Q5VZF2 | MBNL2 | n/a | 0.28 | 745 | 73 | Unclassified |
|  | Muscleblind-like protein 3 | Q9NUK0 | MBNL3 | n/a | 0.28 | 745 | 73 | Unclassified |
|  | Somatostatin receptor type 1 | P30872 | SSTR1 | CHEMBL1917 | 0.28 | 39 | 29 | Membrane receptor |
|  | Somatostatin receptor type 2 | P30874 | SSTR2 | CHEMBL1804 | 0.28 | 39 | 29 | Membrane receptor |
|  | Somatostatin receptor type 4 | P31391 | SSTR4 | CHEMBL1853 | 0.28 | 39 | 29 | Membrane receptor |
|  | Somatostatin receptor type 3 | P32745 | SSTR3 | CHEMBL2028 | 0.28 | 39 | 29 | Membrane receptor |
|  | Somatostatin receptor type 5 | P35346 | SSTR5 | CHEMBL1792 | 0.28 | 39 | 29 | Membrane receptor |
|  | 22 kDa interstitial collagenase | P03956 | MMP1 | CHEMBL332 | 0.28 | 989 | 19 | Metallo Protease |
|  | Stromelysin-1 | P08254 | MMP3 | CHEMBL283 | 0.28 | 989 | 19 | Metallo Protease |
| **cmd3** | Myeloperoxidase | P05164 | MPO | CHEMBL2439 | 0.13 | 2 | 1 | Enzyme |
|  | Thyroid peroxidase | P07202 | TPO | CHEMBL1839 | 0.13 | 2 | 1 | Enzyme |
|  | Eosinophil peroxidase | P11678 | EPX | CHEMBL2438 | 0.13 | 2 | 1 | Enzyme |
|  | Lactoperoxidase | P22079 | LPO | CHEMBL5898 | 0.13 | 2 | 1 | Enzyme |
|  | Peroxidasin homolog | Q92626 | PXDN | n/a | 0.13 | 2 | 1 | Enzyme |
|  | Activation peptide fragment 1 | P00734 | F2 | CHEMBL204 | 0.03 | 715 | 0 | Serine Protease |
|  | Coagulation factor IXa heavy chain | P00740 | F9 | CHEMBL2016 | 0.03 | 744 | 0 | Serine Protease |
|  | Factor X light chain | P00742 | F10 | CHEMBL244 | 0.03 | 744 | 0 | Serine Protease |
|  | Alpha-trypsin chain 1 | P07477 | PRSS1 | CHEMBL209 | 0.03 | 369 | 0 | Serine Protease |
|  | Coagulation factor VII | P08709 | F7 | CHEMBL3991 | 0.03 | 736 | 0 | Serine Protease |
|  | Trypsin-3 | P35030 | PRSS3 | CHEMBL4551 | 0.03 | 369 | 0 | Serine Protease |
|  | Trypsin-2 | P07478 | PRSS2 | CHEMBL3159 | 0.03 | 369 | 0 | Serine Protease |
|  | Urokinase-type plasminogen activator long chain A | P00749 | PLAU | CHEMBL3286 | 0.03 | 196 | 0 | Serine Protease |
|  | Tissue-type plasminogen activator | P00750 | PLAT | CHEMBL1873 | 0.03 | 44 | 0 | Serine Protease |
|  | Hepatocyte growth factor activator long chain | Q04756 | HGFAC | n/a | 0.03 | 196 | 0 | Serine Protease |

^a^ Probability score indicated the target to be a true positive for the ligand.
